# Preventive Efficacy of an Antioxidant Compound on Blood Retinal Barrier Breakdown and Visual Dysfunction in Streptozotocin-Induced Diabetic Rats

**DOI:** 10.3389/fphar.2021.811818

**Published:** 2022-01-03

**Authors:** Alessio Canovai, Rosario Amato, Alberto Melecchi, Massimo Dal Monte, Dario Rusciano, Paola Bagnoli, Maurizio Cammalleri

**Affiliations:** ^1^ Department of Biology, University of Pisa, Pisa, Italy; ^2^ Interdepartmental Research Center Nutrafood “Nutraceuticals and Food for Health”, University of Pisa, Pisa, Italy; ^3^ Research Center, Sooft Italia SpA, Catania, Italy

**Keywords:** oxidative stress, inflammation, gliosis, vasopermeability, apoptosis, electroretinogram

## Abstract

In diabetic retinopathy (DR), high blood glucose drives chronic oxidative stress and inflammation that trigger alterations of the neurovascular balance finally resulting in vascular abnormalities and retinal cell death, which converge towards altered electroretinogram (ERG). In the last years, a growing body of preclinical evidence has suggested that nutrients with anti-inflammatory/antioxidant properties can be able to hamper DR progression since its very early stages. In the present study, we used a streptozotocin-induced rat model of DR, which mimics most aspects of the early stages of human DR, to test the preventive efficacy of a novel compound containing cyanidin-3-glucoside (C3G), verbascoside and zinc as nutrients with antioxidant and anti-inflammatory properties. Western blot, immunofluorescence and electroretinographic analyses demonstrated a dose-dependent inhibition of oxidative stress- and inflammation-related mechanisms, with a significant counterpart in preventing molecular mechanisms leading to DR-associated vasculopathy and its related retinal damage. Preventive efficacy of the compound on dysfunctional a- and b-waves was also demonstrated by electroretinography. The present demonstration that natural compounds, possibly as a consequence of vascular rescue following ameliorated oxidative stress and inflammation, may prevent the apoptotic cascade leading to ERG dysfunction, adds further relevance to the potential application of antioxidants as a preventive therapy to counteract DR progression.

## Introduction

Diabetic retinopathy (DR) is a leading cause of acquired visual impairment in developed countries. DR is characterized by a chronic progression, which might result asymptomatic for several years before the occurrence of overt clinical evidence (Al-Kharashi, 2018). Major signs of DR are morpho functional alterations of retinal vascularization, primarily manifested as a significant increase in vascular leakage, and macular oedema deriving from the blood retinal barrier (BRB) breakdown (Klaassen et al., 2013). Noteworthy, the onset of relevant clinical signs of DR corresponds to advanced stages of the disease that marks a point of no return (Sinclair and Schwartz, 2019). These aspects render the clinical management of DR a challenging topic and feeds the research of treatment approaches to intervene on the early stages of the disease in order to prevent or delay DR progression.

Although the clinical symptoms of DR are almost exclusively attributed to dysfunctional vascularization, DR consists in a complex interplay of pathological processes affecting the integrity of the neurovascular unit since its early stages (Simό et al., 2018; Rossino et al., 2019). In fact, prolonged exposure to high glucose leads to metabolic stress that affects the BRB and the highly specialized neural connections, responsible for vision processes (Feng et al., 2012; Mi et al., 2014). In particular, hyperglycemia initiates an early increase of reactive oxygen species (ROS), which triggers the activation of endogenous antioxidant response through increased levels of nuclear factor erythroid 2-related factor 2 (Nrf2) leading to the transcription of antioxidant enzymes such as heme oxygenase-1 (HO-1) (Kang and Yang, 2020). However, antioxidant defenses are overwhelmed by ROS accumulation thus producing the onset of oxidative stress (Kowluru and Chan, 2007).

Oxidative stress activates inflammation that exacerbates ROS production, both events converging on retinal cell degeneration and altered vascularization (Tang and Kern 2011). Among the several players that participate to the early vascular pathology that characterizes DR, vascular endothelial growth factor (VEGF) and its regulatory transcription factor hypoxia inducible factor-1 (HIF-1) play an important role and their increase takes place in response to hyperglycemia-induced hypoxic environment (Gu et al., 2019). VEGF overexpression induces the loss of tight-junction proteins and subsequent alterations in the BRB integrity (Qaum et al., 2001). Therefore, the early neurovascular dysfunction appears to be strictly related to a series of metabolic unbalances deriving from the hyperglycemic condition of which common denominators are oxidative stress and inflammation (Al-Kharashi, 2018). Their importance along the progression of DR is further corroborated by the fact that strategies already approved for the management of advanced DR, such as treatments with anti-VEGF drugs and the use of steroids, have been found to interfere on inflammatory pathways (Giurdanella et al., 2015; Bucolo et al., 2018). On the other hand, the efficacy of dietary compounds with antioxidant/anti-inflammatory properties has been largely studied for their extensive bioactivity and limited risk of side effects (Rossino and Casini, 2019). However, clinical trials using antioxidants in DR have provided controversial results and much preclinical evidence is still needed to demonstrate the efficacy of antioxidant compounds to counteract the multifactorial nature of DR (Garcia-Medina et al., 2020). In this respect, the use of a multicomponent formula may further improve antioxidant efficacy as the different compounds contained in the mixture may exert multi-target and multifunctional effects by acting at different levels of the same signaling cascade or by modulating different cascades (Leena et al., 2020).

Among natural compounds exerting antioxidant/anti-inflammatory properties, anthocyanin subcomponents, and in particular cyanidins, have been shown to display a good uptake rate, a low decay and a significant clinical relevance, thus resulting one of the most pharmaceutically promising class of nutrients (Khoo et al., 2017). Among cyanidins, cyanidin-3-glucoside (C3G), the most abundant anthocyanin, has a predominant antioxidant capacity that has been reported in several diabetic complications including DR (Sasaki et al., 2007; Wang et al., 2016; Li et al., 2018; Qin et al., 2018).

Additional antioxidant/anti-inflammatory compounds belong to the extensive family of phenylpropanoids, a class of plant-derived polyphenols, which are biosynthesized from the amino acid phenylalanine (Alipieva et al., 2014). Of them verbascoside exerts an anti-inflammatory role due to its ROS scavenging, antioxidant and iron chelating properties (Perron and Brumaghim, 2009; Burgos et al., 2020) although its potential role in DR remains to be established. In addition to plant-derived antioxidant compounds, the activation of enzymes involved in antioxidant defenses has been related to the activity of zinc (Kamińska et al., 2021). Zinc itself is not redox active but is a cofactor that exerts an indirect redox activity by regulating mitochondrial function and, therefore, the rate of ROS generation (Marreiro et al., 2017).

The individual antioxidant efficacy of C3G, verbascoside and zinc has been demonstrated in several experimental models of ocular diseases including DR, glaucoma and age-related macular degeneration (Miao et al., 2013; Chen et al., 2019; Nomi et al., 2019; Oliveira et al., 2020), but information is lacking about their combined efficacy in preventing the early signs of experimental DR.

In the present study, we investigated the efficacy of a compound including C3G, verbascoside and zinc using the chemically induced streptozotocin (STZ) rat model of diabetes, which has been routinely used in preclinical studies and therapeutic drug investigations. This model that mimics the early stages of DR in humans, is characterized by an increased expression of oxidative stress and inflammation markers resulting in VEGF-induced retinal vasopermeability that causes BRB breakdown and retinal dysfunction (Naderi et al., 2019). In STZ rats, we evaluated the preventive efficacy of the compound on oxidative stress, inflammation, gliotic responses and apoptotic markers. In addition, its preventive efficacy on neuroretinal components responsible for vision processes was also assessed by electroretinography.

## Materials and Methods

### Reagents

Rabbit polyclonal anti-NRF2 (catalog n. ab92946), rabbit polyclonal anti-HO-1 (catalog n. ab13243), rabbit polyclonal anti-NF-kB p65 (catalog n. ab16502), rabbit monoclonal anti-HIF-1α (catalog n. ab179483), rabbit monoclonal anti-Bax (catalog n. ab182733), rabbit polyclonal anti-Bcl-2 (catalog n. ab194583), rabbit polyclonal anti-cleaved caspase 3 (catalog n. ab2302), rabbit monoclonal anti-GFAP (catalog n. ab207165), goat polyclonal anti-rabbit conjugated with either Alexa-Fluor 555 (catalog n. ab150078) or Alexa-Fluor 488 (catalog n. ab150077) antibodies were purchased from Abcam (Cambridge, United Kingdom). Rabbit polyclonal anti-pNF-kB p65 (Ser 536) (catalog n. sc-33020), mouse monoclonal anti-IL-6 (catalog n. sc-57315), rabbit polyclonal anti-VEGF (catalog n. sc-507) antibodies were purchased from Santa Cruz Biotechnology, Inc (Dallas, TX, United States). Rabbit polyclonal anti-ZO-1 (catalog n. 40-2200), mouse monoclonal anti-Claudin 5 (catalog n. 35-2500) antibodies were purchased from Invitrogen (Waltham MA, United States). Mouse monoclonal anti-β-actin (catalog n. A2228), rabbit polyclonal anti-mouse HRP-conjugated (catalog n. A9044) antibody were purchased from Sigma-Aldrich (St. Louis, MO, United States). Rabbit monoclonal anti-cleaved caspase 3 (catalog n. 9664S) antibody used for immunofluorescence was purchased from Cell Signaling Technology (Danvers, MA, United States). Goat polyclonal anti-rabbit HRP-conjugated (catalog n. 170-6515) antibody was purchased from Bio-Rad Laboratories, Inc (Hercules, CA, United States).

### Animals

Animals were managed in accordance with the Association for Research in Vision and Ophthalmology statement for the Use of Animals in Ophthalmic and Vision Research. The present study is also in agreement with the European Communities Council Directive (2010/63/UE) and the Italian guidelines for animal care (DL 26/14). The experimental protocol was authorized by the Commission for Animal Wellbeing of the University of Pisa (protocol no. 133/2019-PR, February 14, 2019). Efforts to reduce both the number and suffering of the animals were made in accordance with the 3Rs principles for ethical use of animals in scientific research. Male Sprague Dawley rats (8 weeks old, about 200 g weight) were purchased from Envigo Italy (San Pietro al Natisone, Italy). Animals were housed in a regulated environment (23 ± 1°C, 50 ± 5% humidity) with 12 h light/dark cycles (lights on at 08:00 a.m.) and fed with a standard diet and water ad libitum. Thirty-two rats were used. They were divided in four groups of eight rats each: control, STZ untreated, STZ-treated with the low dose of the mixture and STZ-treated with the high dose (see below). To evaluate the effect of the compound on weight, glycemia and visual function of healthy rats, additional six rats were supplemented with the compound.

### STZ-Induced Model of Diabetes and Treatments

STZ (Sigma-Aldrich, St. Louis, MO, United States) diluted in citrate buffer, pH 4.5, was intraperitoneally injected at 65 mg/kg. Age-matched rats treated with vehicle were considered as the control group. Blood glucose was measured 3 days after the injection by tail sampling using a OneTouch Ultra glucometer (LifeScan Inc, Milpitas, CA, United States) to confirm the diabetic induction. Animals were considered diabetic if glycemia was ≥250 mg/dl. Blood glucose level was then regularly checked once a week for the entire experimental period. Among diabetic rats, 16 animals for each group were treated with a compound including *Oryza sativa* L. seeds (C3G titrated at 20%), *Verbascum thapsus* L (verbascoside titrated at 10%) and zinc gluconate (zinc titrated at 13.23%) with a content ratio of 60:30:10, respectively. The compound was diluted in water at 84 mg/ml and administered at low dose (100 µL containing 1.0 mg of C3G, 0.25 mg of verbascoside and 0.125 mg of zinc) or high dose (300 µL containing 3.0 mg of C3G, 0.75 mg of verbascoside and 0.375 mg of zinc). Dosage range corresponds to that recommended in humans, normalized by the body surface area for interspecies drug dosage translation (Nair and Jacob, 2016). Rats were treated once daily by oral gavage for 30 days after STZ administration. Thirty days after STZ administration, the animals underwent to electroretinography. In Table 1, the experimental groups, the schedule of treatment, the amount of daily administered components and the number of rats for each experimental group are shown.

**TABLE 1:** Experimental groups and schedule of treatments.

Group	N	Treatment	Daily amount	Days of treatment
Control	8	None	—	—
	3	+ low dose	C3G (1.0 mg), verbascoside (0.25 mg), zinc (0.125 mg)	30
	3	+ high dose	C3G (3.0 mg), verbascoside (0.75 mg), zinc (0.375 mg)	30
STZ	8	None	—	—
	8	+ low dose	C3G (1.0 mg), verbascoside (0.25 mg), zinc (0.125 mg)	30
	8	+ high dose	C3G (3.0 mg), verbascoside (0.75 mg), zinc (0.375 mg)	30

### Electroretinogram

In the four groups, retinal function was examined with scotopic full-field electroretinogram (ERG). Before ERG, rats underwent overnight dark-adaptation and subsequent anesthesia with intraperitoneal injection of 30 mg/kg sodium pentobarbital. Pupils were dilated with a topical drop of 1% tropicamide (Allergan S.p.A.) and the body temperature was kept constantly at 37.5°C by a heating pad. The electrophysiological signals were recorded using silver/silver chloride corneal ring electrodes inserted under the lower eyelids to avoid visual field obstruction. In order to prevent dryness and clouding of the ocular surface, saline solution drops were intermittently instilled. Each corneal electrode was referred to a needle electrode inserted subcutaneously at the level of the corresponding frontal region, and the ground electrode was inserted subcutaneously at the tail root. Scotopic ERG, which primarily measures rod function, was evoked by flashes of increasing light intensities ranging from −3.4 to one log cd-s/m^2^ generated through a Ganzfeld stimulator (Biomedica Mangoni, Pisa, Italy). An interval of 20 s between light flashes was adjusted to allow response recovery. Scotopic responses were collected simultaneously from both eyes, amplified at 10000x gain, filtered with a 0.2–500 Hz bandpass and digitized at 5 kHz rate with a data acquisition device (Biomedica Mangoni). ERG waveforms were analyzed using a customized program (Biomedica Mangoni). In the absence of light stimulation, the electrical activity was measured to evaluate noise amplitude. In accordance with the International Society for Clinical Electrophysiology guidelines, the a-wave amplitude was measured from the pre-stimulus baseline to the negative through of the a-wave while the b-wave amplitude was retrieved from the through of the a-wave to the peak of the b-wave. Data were pooled and reported as mean amplitude ±SEM (in μV). Intensity-response function of the b-wave was fitted to the following modified Naka-Rushton function (Naka and Rushton, 1966):

$$V\left(I\right)=V0+\frac{V\max\;\times In}{In+kn}$$

In this equation, V is the amplitude of the b-wave (in μV), I is the stimulus intensity (in log cd-s/m^2^), V0 is the nonzero baseline effect, Vmax is the saturated amplitude of the b-wave (in μV) and k is the stimulus intensity that evokes b-waves of half-maximum amplitude (in log cd-s/m^2^); n, which was constrained to unity, is a dimensionless constant that controls the slope of the function and represents the degree of heterogeneity of retinal sensitivity.

### Detection of Vascular Leakage by Evans Blue Dye Perfusion

After electroretinography, two rats per group were anesthetized with an intraperitoneal injection of 30 mg/kg sodium pentobarbital and perfused through the left ventricle with 0.5% Evans blue dye (Sigma-Aldrich) in phosphate-buffer saline (PBS) that was allowed to circulate for 10 min. The animals were then sacrificed, and retinas were dissected and flat mounted onto microscope slides. Retinas were then examined by an epifluorescence microscope (Ni-E; Nikon Europe, Amsterdam, Netherlands) and the images were acquired using a 10x plan apochromat objective and a digital camera (DS-Fi1c camera; Nikon-Europe).

### Western Blot

In six rats per group, eyes were enucleated and alternatively used for Western blot analysis in explanted retinas or immunohistochemistry (see below). For Western blot, retinas were lysed with RIPA lysis buffer (Santa Cruz Biotechnology, Dallas, TX, United States) implemented with phosphatase and proteinase inhibitor cocktails (Roche Applied Science, Indianapolis, IN, United States). Protein content was evaluated by Micro BCA protein assay (Thermo Fisher Scientific, Waltham, MA, United States). Fourty micrograms of proteins per sample were separated by SDS-PAGE (4–20%; Bio-Rad Laboratories, Inc., Hercules, CA, United States) and gels were subsequently transblotted onto nitrocellulose membranes (Bio-Rad Laboratories, Inc.). Membranes were blocked with 5% skim milk for 1 h at room temperature and then incubated overnight at 4°C with the solutions of primary anti-NRF2 (1:1,000), anti-HO-1 (1:500), anti-pNF-kB p65 (Ser 536) (1:100), anti-NF-kB p65 (1:1,000), anti-IL-6 (1:100), anti-HIF-1α (1:1,000), anti-VEGF (1:100), anti-ZO-1 (1:500), anti-Claudin 5 (1:500), anti-Bax (1:500), anti-Bcl-2 (1:500), anti-cleaved caspase 3 (1:500), anti-β-actin (1:2500) antibodies. Thereafter, membranes were incubated for 2 h at room temperature with appropriate HRP-conjugated secondary anti-mouse or anti-rabbit (1:5,000) antibodies. Blots were developed using the Clarity western enhanced chemiluminescence substrate (Bio-Rad Laboratories, Inc.) and the images were acquired by the ChemiDoc XRS+ (Bio-Rad Laboratories, Inc.). The optical density (OD) relative to the target bands (Image Lab 3.0 software; Bio-Rad Laboratories, Inc.) was normalized to the corresponding OD of β-actin as loading control or nuclear factor kappa-light-chain-enhancer of activated B cells (NF-kB) p65 as appropriate.

### Measurement of ROS Levels

ROS levels were measured using the general oxidative stress probe 2′,7′-dichlorodihydrofluorescein diacetate (DCFH-DA) (Invitrogen, CA, United States). DCFH-DA, a nonfluorescent dye, is cleaved by esterase activity to yield dichlorodihydrofluorescein (DCFH), which is subsequently oxidized by ROS to form fluorescent dichlorofluorescein (DCF). Retinal samples containing 20 μg proteins were incubated with 50 μM DCFHDA in 96-well plates. After 60 min at 37°C, fluorescence intensity was detected over 60 min using a microplate reader (FLUOstar Omega, BMG Labtech, Ortenberg, Germany) at excitation 488 nm and emission 525 nm. The relative ROS levels were expressed as arbitrary fluorescence units per μg of protein.

### Immunofluorescence

Enucleated eyes were immersion-fixed in 4% paraformaldehyde in 0.1 M PBS for 2 h at room temperature. Fixed eyes were transferred to 25% sucrose in 0.1 M PBS and stored at 4°C. Following the inclusion in cryo-gel medium, fixed samples were cut into 10 µm thick coronal sections and mounted onto glass slides. Mounted sections were then incubated with the solutions of primary anti-HO-1 (1:500), anti-GFAP (1:400) or anti-cleaved caspase 3 (1:400) antibodies diluted in 0.1 M PBS containing 0.1% v/v Triton X-100, overnight at 4°C. Mounted sections were incubated with goat polyclonal anti-rabbit secondary antibodies conjugated with Alexa-Fluor 555 (ab150078, Abcam, Cambridge, United Kingdom; dilution: 1:200) or Alexa-Fluor 488 (ab150077, Abcam; dilution: 1:200) diluted in 0.1 M PBS containing 0.1% v/v Triton X-100 for 2 h at room temperature. Finally, retinal sections were coverslipped with Fluoroshield mounting medium containing 4′, 6-diamidino-2-phenylindole (DAPI; Abcam). In order to analyze the outer BRB, the retinal pigmented epithelium (RPE)-choroid complexes were isolated from fixed eyes and incubated for 72 h at 4°C in anti-ZO-1 antibody (1:100 in 0.1 M PB containing 1.0% Triton X-100). Subsequently, they were incubated for 48 h at 4 °C in anti-rabbit secondary antibody conjugated with Alexa-Fluor 488 (1:200) followed by 0.1 M PB rinsing. Four radial incisions were made in the RPE-choroid complexes that were flat-mounted on gelatin-coated glass slides. Images of retinal sections or RPE-choroid flatmounts were acquired through an epifluorescence microscope (Nikon-Europe) at 20× and 40x, respectively using a digital camera (Nikon-Europe). The quantification of the GFAP immunostaining was performed by averaging the fluorescence intensity of five coronal sections (4 images per section) randomly chosen from each retina (6 retinas per group). Fluorescence intensity was calculated on grayscale images normalized for the background by measuring the mean gray level using the analysis tool of Adobe Photoshop. The thickness of the outer nuclear layer (ONL) was measured as the interface between the outer plexiform layer and photoreceptor inner segment, while the thickness of the inner nuclear layer (INL) was measured as the interface between the outer plexiform layer and the inner plexiform layer. The quantification of ONL and INL thickness was performed by averaging measurements from five coronal sections (4 images per section) randomly chosen from each retina (6 retinas per group).

### Statistical Analysis

Graph Pad Prism 8.0.2 software (Graph-Pad Software, Inc., San Diego, CA, United States) was used for the statistical analyses. Differences among groups were assessed through one-way or two-ways ANOVA followed by Tukey’s or Bonferroni’s multiple comparison post hoc test, respectively. Differences with *p* < 0.05 were considered significant. All data are expressed as means ± SEM of the indicated n values.

## Results

### The Compound Does Not Affect Body Weight and Glycemia

**FIGURE 1:**
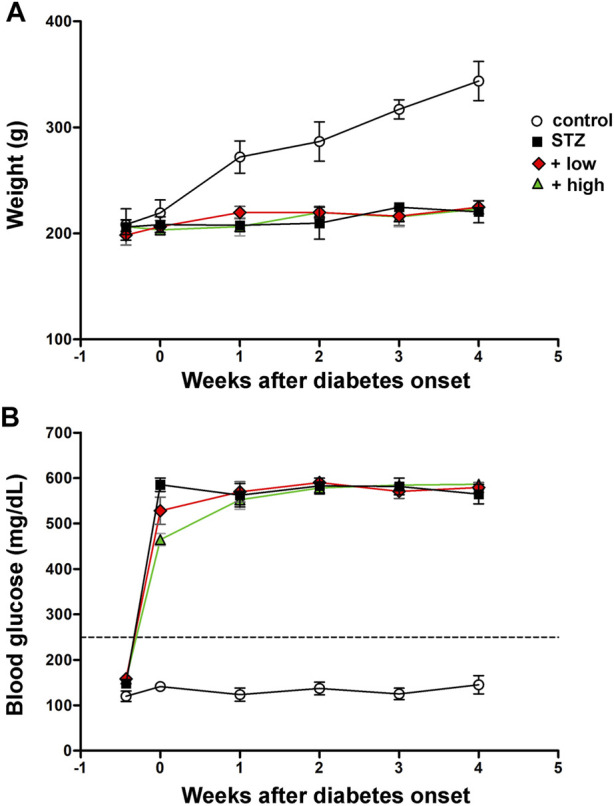
Longitudinal evaluation of body weight **(A)** and blood glucose **(B)** in controls, STZ-untreated rats or STZ rats treated with either the low dose (C3G, 1.0 mg; verbascoside, 0.25 mg and zinc, 0.125 mg) or the high dose (C3G, 3.0 mg; verbascoside, 0.75 mg and zinc, 0.375 mg) of the compound. Data are expressed as mean ± SEM. Statistical significance was assessed by two-ways ANOVA followed by Bonferroni’s multiple comparison post-hoc test (N = 8).

Control rats displayed a significant age-dependent gain of weight. In line with previous findings (Furman, 2015), STZ injection resulted in markedly lower body weight as compared to controls (*p* < 0.01, Figure 1A). Following STZ administration, blood glucose levels were significantly increased and remained higher than in controls until the animals underwent to ERG recordings and were subsequently sacrificed (*p* < 0.0001 vs control, Figure 1B). Blood glucose levels did not significantly differ between STZ rats either untreated or treated with the compound at both doses. Control rats treated with the compound displayed an age-dependent gain of weight and blood glucose levels comparable to those of untreated controls (Supplementary Figure S1).

### The Compound Protects the Retina From Oxidative Stress and Inflammation

**FIGURE 2:**
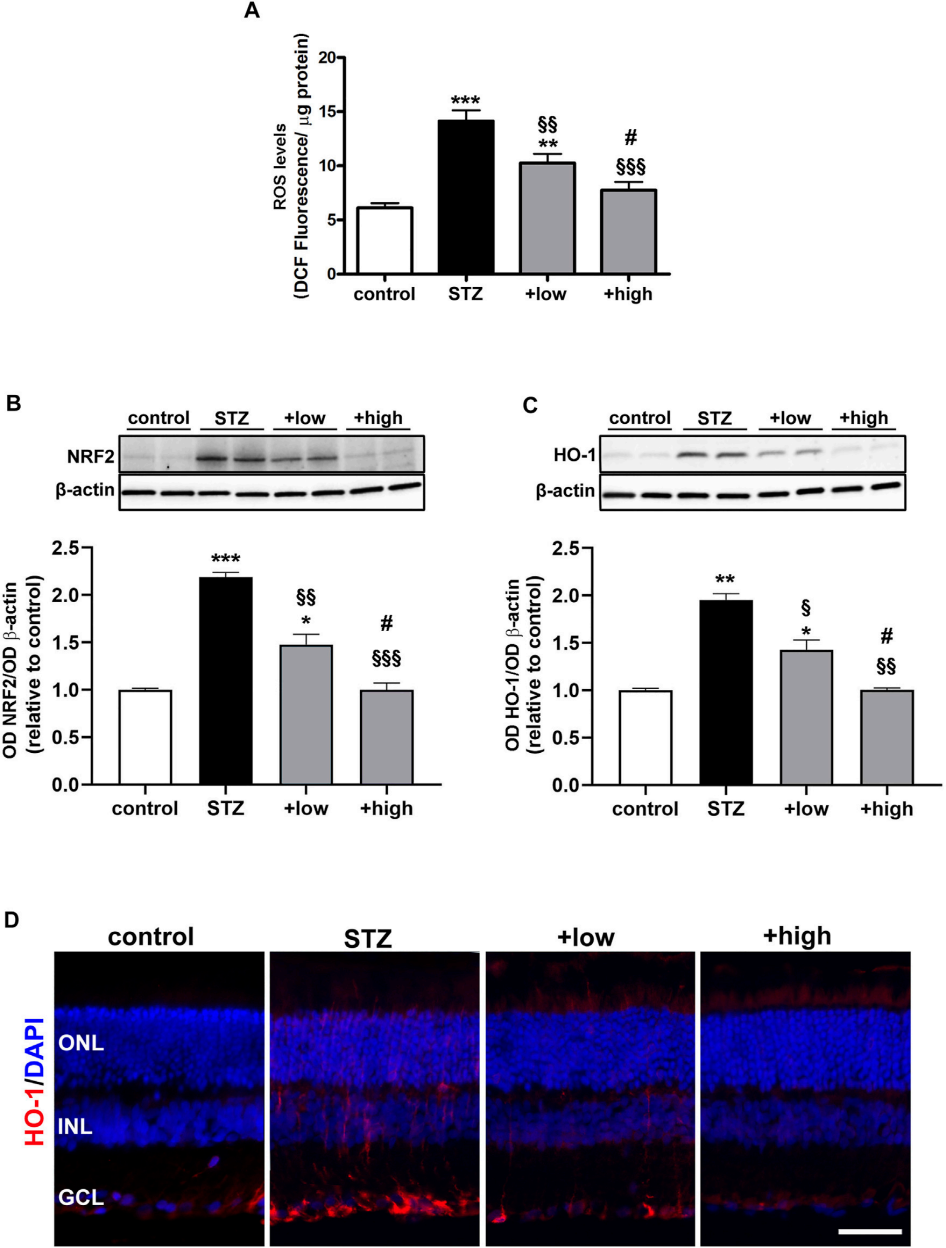
Effects of the compound on ROS levels and markers of oxidative stress **(A)** ROS levels in controls, STZ untreated or STZ treated with either the low dose (C3G, 1.0 mg; verbascoside, 0.25 mg and zinc, 0.125 mg) or the high dose (C3G, 3.0 mg; verbascoside, 0.75 mg and zinc, 0.375 mg) of the compound. Representative Western blots and densitometric analysis of NRF2 **(B)** and HO-1 **(C)** in controls, STZ untreated or STZ rats treated with either the low dose or the high dose. β-actin was used as loading control. Data are expressed as mean ± SEM. Statistical significance was assessed by one-way ANOVA followed by Tukey’s multiple comparison post-hoc test (N = 6). ^*^ *p* < 0.05, ^**^ *p* < 0.01 and ^***^ *p* < 0.001 vs control; ^§^ *p* < 0.05, ^§§^ *p* < 0.01 and ^§§§^ *p* < 0.001 vs STZ; ^#^ *p* < 0.05 vs low dose-treated STZ **(D)** Representative images of retinal cross sections immunolabeled for HO-1 (red) and counterstained with DAPI (blue). Scale bar, 50 μm. GCL, ganglion cell layer; INL, inner nuclear layer; ONL, outer nuclear layer.

The preventive efficacy of the compound was tested on ROS generation. Levels of specific markers of oxidative stress and inflammation known to play a crucial role in the early progression of DR (Al-Kharashi, 2018) were also measured. As shown in Figure 2A,**,** STZ rats displayed a significant increase in ROS generation as compared to controls (*p* < 0.001 vs control). STZ rats treated with the compound at low dose showed lower levels of ROS as compared to untreated STZ (*p* < 0.01 vs STZ), although still resulting significantly higher than in controls (*p* < 0.01 vs control). In STZ rats treated with the compound at high dose, ROS levels were comparable to those measured in controls (*p* > 0.05 vs control). Oxidative stress was evaluated by analyzing the protein levels of NRF2, a ROS-sensitive transcriptional factor, and HO-1, one of the antioxidant enzymes involved in defensive responses to oxidative stress (Kang and Yang., 2020). As shown in Figures 2B, C, the densitometric analysis of immunoblots revealed a significant increment in both Nrf2 and HO-1 in STZ rats as compared to controls (*p* < 0.01 vs control). This increment was partially attenuated by the compound at low dose (*p* < 0.05 vs STZ), but completely prevented by the high dose (*p* < 0.001 vs STZ; *p* < 0.05 vs low dose). As shown in Figure 2D, immunofluorescence analysis revealed a faint HO-1 immunoreactivity in control retinas mainly confined to the ganglion cell layer (GCL). In retinas of STZ rats, HO-1 immunoreactivity was significantly increased in the GCL and expanded toward the outer retina, clearly depicting vertical processes in the inner plexiform layer (IPL) and cellular profiles localized in the inner nuclear layer (INL). Retinas of rats treated with compound at low dose displayed a less evident HO-1 immunostaining as compared to untreated STZ rats, with rare spots in the GCL and residual labeling in the IPL and INL. HO-1 immunostaining was not detectable in the IPL and INL following the treatment with high dose, while a basal labeling was observed in the GCL similarly to what found in the control retina.

**FIGURE 3:**
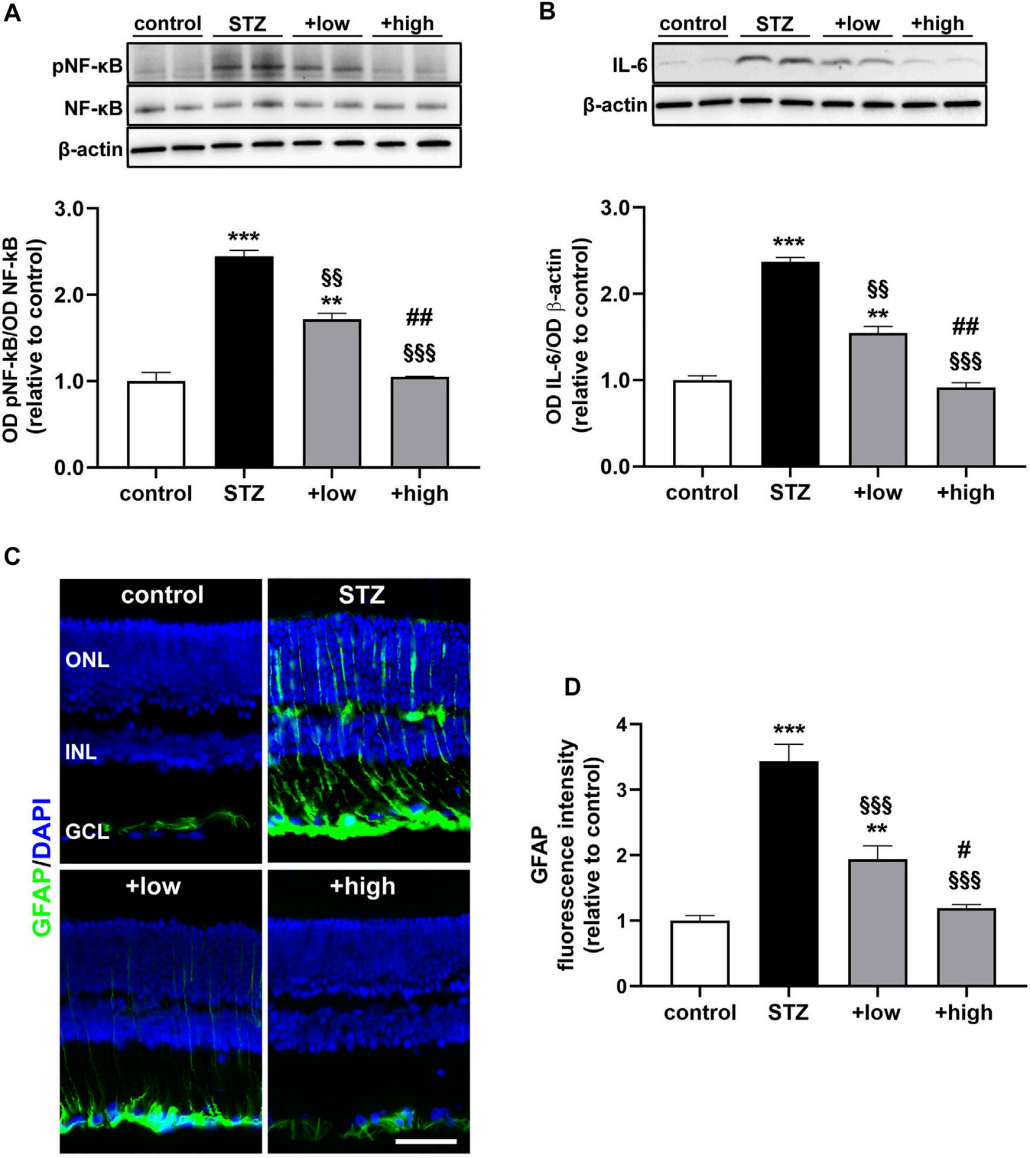
Effects of the compound on inflammatory and gliosis markers. Representative Western blots and densitometric analysis of levels of pNF-kB **(A)** and IL-6 **(B)** in controls, STZ untreated or STZ rats treated with either the low dose (C3G, 1.0 mg; verbascoside, 0.25 mg and zinc, 0.125 mg) or the high dose (C3G, 3.0 mg; verbascoside, 0.75 mg and zinc, 0.375 mg) of the compound. Levels of pNF-kB were normalized to NF-kB levels, while IL-6 was normalized to the loading control β-actin. Data are expressed as mean ± SEM. Statistical significance was assessed by one-way ANOVA followed by Tukey’s multiple comparison post-hoc test (N = 6). ^**^ *p* < 0.01 and ^***^ *p* < 0.001 vs control; ^§§^ *p* < 0.01 and ^§§§^ *p* < 0.001 vs STZ; ^##^ *p* < 0.01 vs low dose treated STZ **(C)** Representative images of retinal cross sections immunolabeled for GFAP (green) and counterstained with DAPI (blue). Scale bar, 50 μm. GCL, ganglion cell layer; INL, inner nuclear layer; ONL, outer nuclear layer **(D)** Quantitative analysis of GFAP immunofluorescence intensity. Data are expressed as mean ± SEM. Statistical significance was assessed by one-way ANOVA followed by Tukey’s multiple comparison post-hoc test (N = 6). ^**^ *p* < 0.01 and ^***^ *p* < 0.001 vs control; ^§§^ *p* < 0.01 and ^§§§^ *p* < 0.001 vs STZ; ^#^ *p* < 0.05 and ^##^ *p* < 0.01 vs low dose treated STZ.

The protein levels of the phosphorylated form of the p65 subunit of NF-kB, a master transcriptional regulator of pro-inflammatory factors and interleukin 6 (IL-6), a related pro-inflammatory cytokine (Liu et al., 2017), were also measured. As shown in Figures 3A, B, STZ rats displayed a marked increase in pNF-KB and IL-6 as compared to controls (*p* < 0.001 vs control). STZ rats treated with the compound at low dose showed lower levels of both markers as compared to untreated STZ (*p* < 0.01 vs STZ), although still resulting significantly higher than in controls (*p* < 0.01 vs control). On the other hand, in STZ rats treated with the high dose, the levels of pNF-kB and IL-6 were comparable to those measured in controls (*p* > 0.05 vs control). The activation of inflammatory processes triggers glial reactivity, which participates to the chronic inflammatory response (Rübsam et al., 2018). The reactive phenotype of glial cells was analyzed by immunostaining with glial fibrillary acidic protein (GFAP), a well-established marker of gliosis (Figure 3C). In control retinas, basal GFAP labeling was confined to the GCL. In contrast, STZ retinas showed an evident increment of GFAP immunoreactivity in the GCL together with densely immunopositive processes spreading across retinal layers as a typical hallmark of Müller cell reactivity. Comparable immunostaining could be detected in retinas of low dose-treated rats, although GFAP immunoreactivity was less prominent both in the GCL and in Müller cell processes. On the other hand, retinas of rats treated with the high dose displayed a GFAP immunostaining almost similar to that of controls, with the usual basal staining and barely detectable immunoreactive vertical processes. As shown in Figure 3D, quantitative analysis of fluorescence intensity showed a marked increment in GFAP immunoreactivity in STZ rat as compared to controls (*p* < 0.001). In STZ rats treated with the low dose, GFAP immunoreactivity was significantly lower than in untreated STZ rats (*p* < 0.001 vs STZ), while STZ rats treated with the high dose displayed GFAP immunofluorescence intensity comparable to that of controls (*p* > 0.05 vs control).

### VEGF-Induced Vascular Permeability and BRB Breakdown Are Prevented by the Compound

**FIGURE 4:**
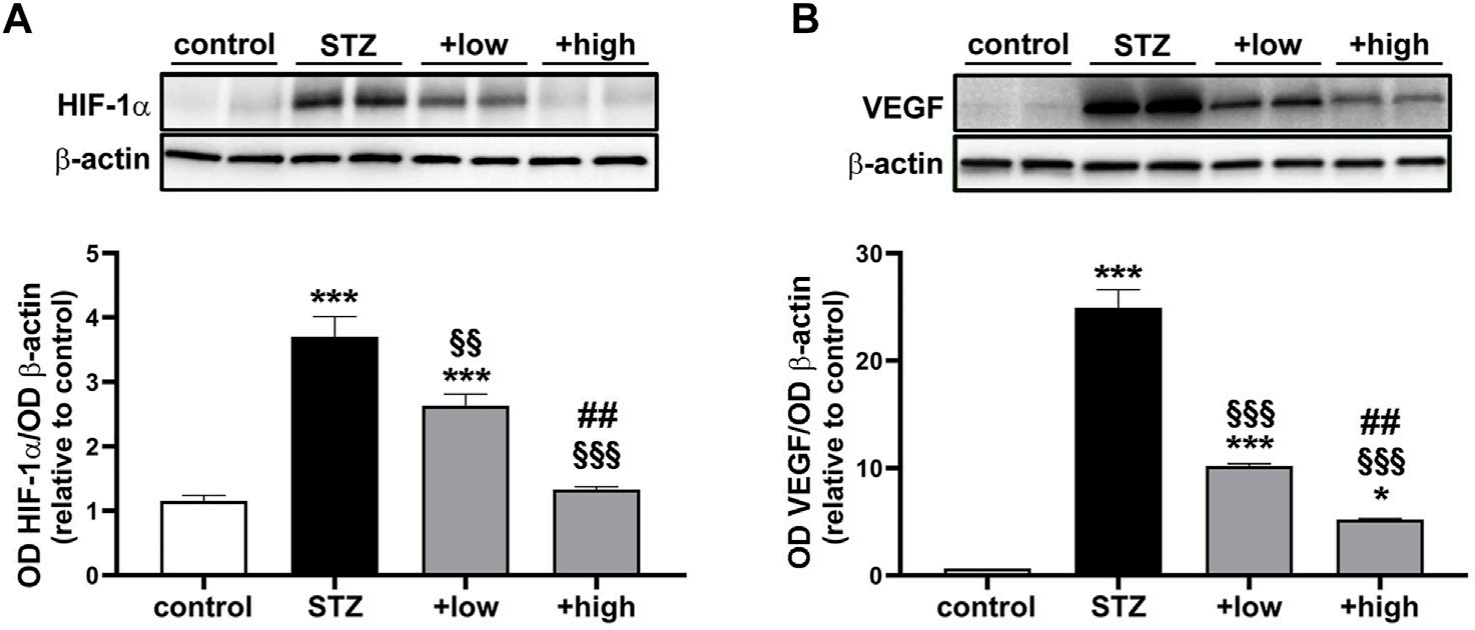
Effects of the compound on vascular-related markers. Representative Western blots and densitometric analysis of HIF-1α **(A)** and VEGF **(B)** in controls, STZ untreated or STZ rats treated with either the low dose (C3G, 1.0 mg; verbascoside, 0.25 mg and zinc, 0.125 mg) or the high dose (C3G, 3.0 mg; verbascoside, 0.75 mg and zinc, 0.375 mg) of the compound. β-actin was used as loading control. Data are expressed as mean ± SEM. Statistical significance was assessed by one-way ANOVA followed by Tukey’s multiple comparison post-hoc test (N = 6). ^*^ *p* < 0.05 and ^***^ *p* < 0.001 vs control; ^§§^ *p* < 0.01 and ^§§§^ *p* < 0.001 vs STZ; ^##^ *p* < 0.01 vs low dose treated STZ.

Oxidative and inflammatory processes have a direct impact on molecular mechanisms regulating the vascular homeostasis through the alteration of the HIF-1-dependent pathway and the consequent dysregulation of angiogenic factors such as VEGF (Semeraro et al., 2015). As shown in Figure 4, HIF-1α levels were significantly increased in STZ rats as compared to controls (*p* < 0.0001). The increment in HIF-1α was significantly attenuated by the compound in a dose-dependent fashion (low dose *p* < 0.001 vs STZ; high dose *p* < 0.0001 vs STZ), with the high dose maintaining HIF-1α to control levels (*p* > 0.05 vs control; Figure 4A). Similarly, STZ rats displayed a marked increase in VEGF levels as compared to controls (*p* < 0.0001). The administration of the compound dose-dependently prevented VEGF accumulation with increased efficacy of the high dose (*p* < 0.0001 vs low dose), although VEGF levels were still higher than in controls (high dose *p* < 0.05 vs control; Figure 4B).

**FIGURE 5:**
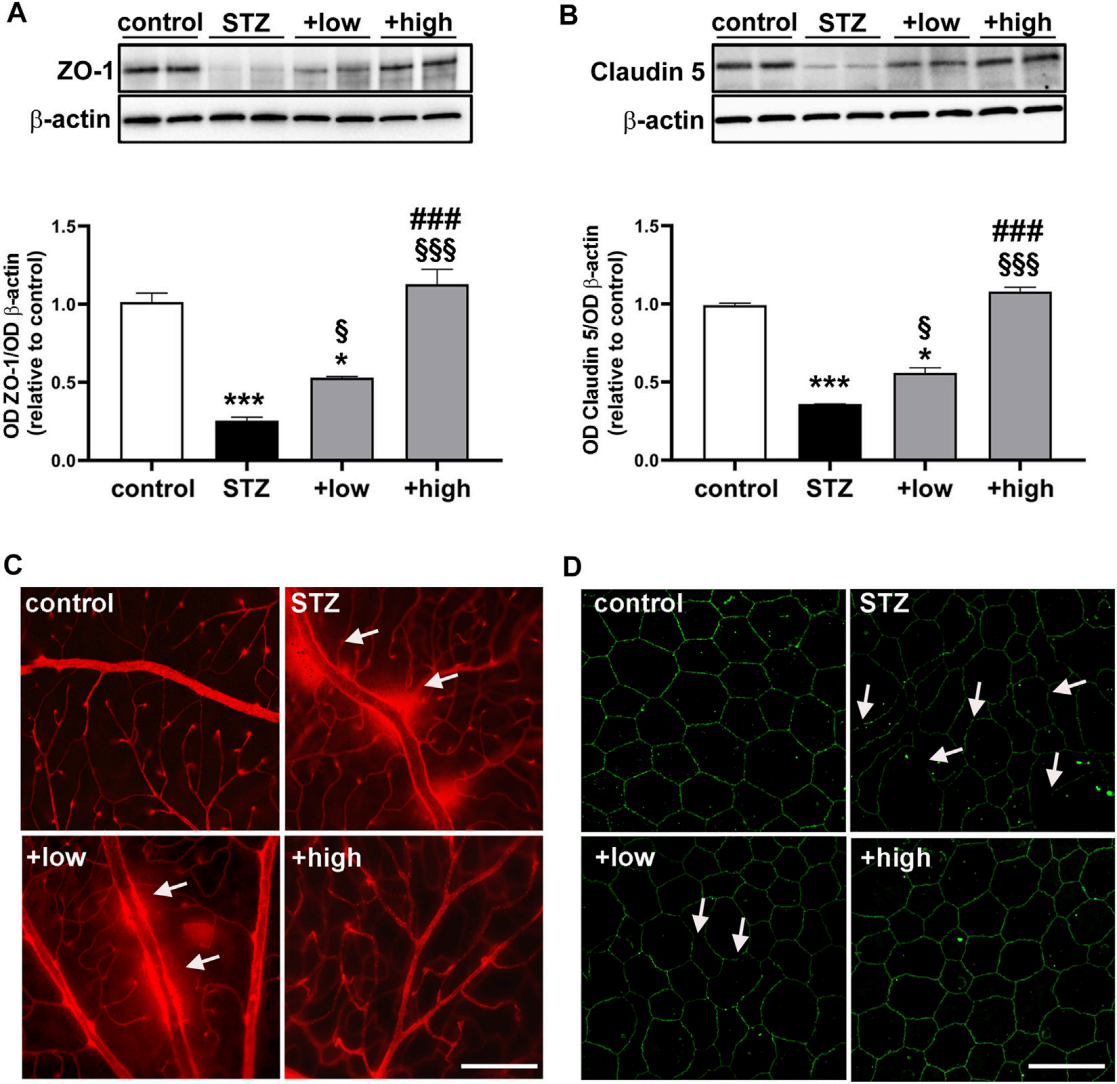
Effects of the compound on BRB markers and vascular leakage. Representative Western blots and densitometric analysis of ZO-1 **(A)** and Claudin 5 **(B)** in controls, STZ untreated or STZ rats treated with either the low dose (C3G, 1.0 mg; verbascoside, 0.25 mg and zinc, 0.125 mg) or the high dose (C3G, 3.0 mg; verbascoside, 0.75 mg and zinc, 0.375 mg) of the compound. β-actin was used as loading control. Data are expressed as mean ± SEM. Statistical significance was assessed by one-way ANOVA followed by Tukey’s multiple comparison post-hoc test (N = 6). ^*^ *p* < 0.05 and ^***^ *p* < 0.001 vs control; ^§^ *p* < 0.05 and ^§§§^ *p* < 0.001 vs STZ; ^###^ *p* < 0.001 vs low dose treated STZ **(C)** Representative images of whole-mounted retinas after Evans blue dye perfusion. Vascular leakage is indicated by the white arrows. Scale bar, 200 μm **(D)** Representative images of RPE-choroid flatmounts stained with ZO-1. White arrows indicate large holes appeared between the RPE cells Scale bar, 20 μm.

BRB dysfunction resulting from altered HIF-1α-VEGF axis was evaluated by analyzing the levels of zonula occludens 1 (ZO-1) and Claudin 5 as components of the inter-endothelial tight junctions. The BRB integrity was also assessed with the Evans Blue dye perfusion of retinal vessels (for inner BRB) and with ZO-1 immunostaining in RPE-choroid flatmounts (for outer BRB). As shown in Figures 5A, B, the levels of ZO-1 and Claudin five were drastically decreased in STZ retinas (*p* < 0.0001 vs control). The treatment with the compound was found to prevent protein loss with dose-dependent efficacy (low dose *p* < 0.05 vs STZ; high dose *p* < 0.0001 vs STZ), with the high dose displaying ZO-1 and Claudin five levels comparable to those of controls (*p* > 0.05 vs control). As shown in Figure 5C, in STZ rats, the dysregulation of BRB markers was correlated with inner BRB breakdown. In fact, Evans blue, a dye that binds to plasma proteins, was restricted to the vascular lumen in control retinas. Contrariwise, several focal points of extravasation were visible in STZ retinas. The vascular leakage was still evident in STZ rats treated with the compound at low dose, while the extravasation appeared more contained or even absent in retinas of rats treated with the high dose. ZO-1 immunostaining in RPE-choroid flatmounts from STZ rats revealed the presence of large holes between RPE cells indicating a significant loss of tight junctions leading to outer BRB breakdown (Figure 5D). Supplementation with the compound dose-dependently prevented tight junction loss with no apparent differences between controls and STZ rats treated with the high dose.

### The Compound Protects the Retina From Apoptosis and ERG Dysfunction

**FIGURE 6:**
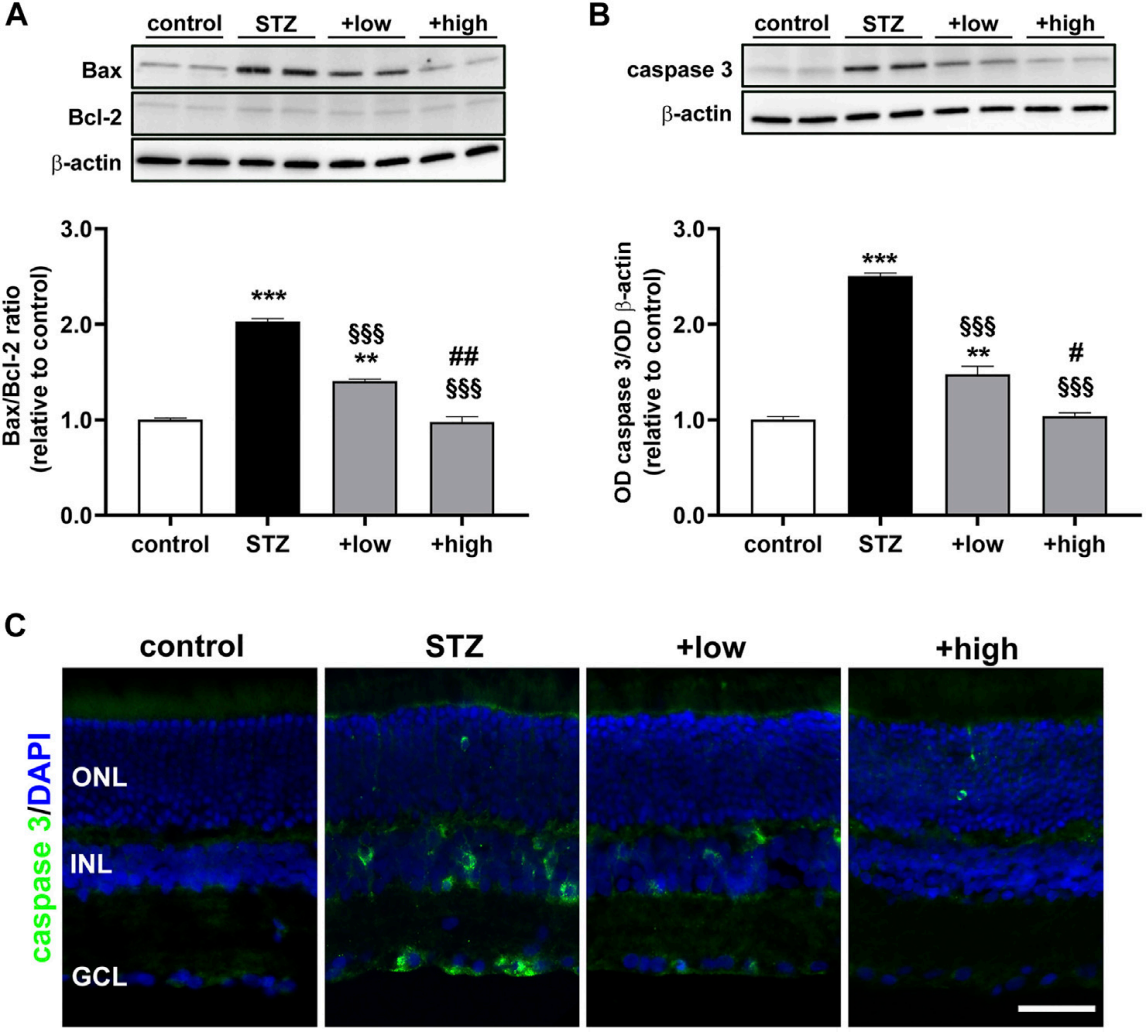
Effects of the compound on apoptotic markers. Representative Western blots and densitometric analysis of Bax/Bcl-2 ratio **(A)** and cleaved caspase 3 **(B)** in controls, STZ untreated or STZ rats treated with either the low dose (C3G, 1.0 mg; verbascoside, 0.25 mg and zinc, 0.125 mg) or the high dose (C3G, 3.0 mg; verbascoside, 0.75 mg and zinc, 0.375 mg) of the compound. The levels of cleaved caspase 3 were normalized to the loading control β-actin. Data are expressed as mean ± SEM. Statistical significance was assessed by one-way ANOVA followed by Tukey’s multiple comparison post-hoc test (N = 6). ^**^ *p* < 0.01 and ^***^ *p* < 0.001 vs control; ^§§§^ *p* < 0.001 vs STZ; ^#^ *p* < 0.05 and ^##^ *p* < 0.01 vs low dose treated STZ **(C)** Representative images of retinal cross sections immunolabeled for cleaved caspase 3 (green) and counterstained with DAPI (blue). Scale bar, 50 μm. GCL, ganglion cell layer; INL, inner nuclear layer; ONL, outer nuclear layer.

A growing body of evidence has underlined that early DR is characterized by apoptosis-related degenerative processes and vascular abnormalities that concur to ERG dysfunction (Barber and Baccouche, 2017). Whether the compound might influence the levels of proapoptotic markers was assessed by evaluating the ratio of pro-apoptotic Bax to anti-apoptotic Bcl-2 proteins as a major checkpoint in the apoptotic pathway. Downstream to the Bax/Bcl-2 ratio, levels of activated caspase 3 as the major effector protease driving the programmed cell death were also determined. As shown by the representative blots and the densitometric analysis in Figures 6A, B, the Bax/Bcl-2 ratio and the levels of caspase 3 were significantly increased in STZ rats as compared to controls (*p* < 0.0001). Treatment with the compound dose-dependently prevented the STZ-induced increase in Bax/Bcl-2 ratio and caspase 3, with a partial efficacy of the low dose (*p* < 0.001 vs STZ), while their increase was completely prevented by the high dose (*p* < 0.0001 vs STZ; *p* > 0.05 vs control). The evidence of attenuated levels of caspase 3 was further supported by immunofluorescence analysis (Figure 6C). In STZ rats, increased caspase 3 was localized to cellular profiles in the GCL and INL as compared to controls in which caspase 3 immunostaining was absent. After the low dose, caspase 3-immunopositive cells were less evident and faintly stained, although some cellular profiles localized to the GCL and INL were still visible. Conversely, no caspase 3-immunopositive cell profiles could be observed after the high dose.

**FIGURE 7:**
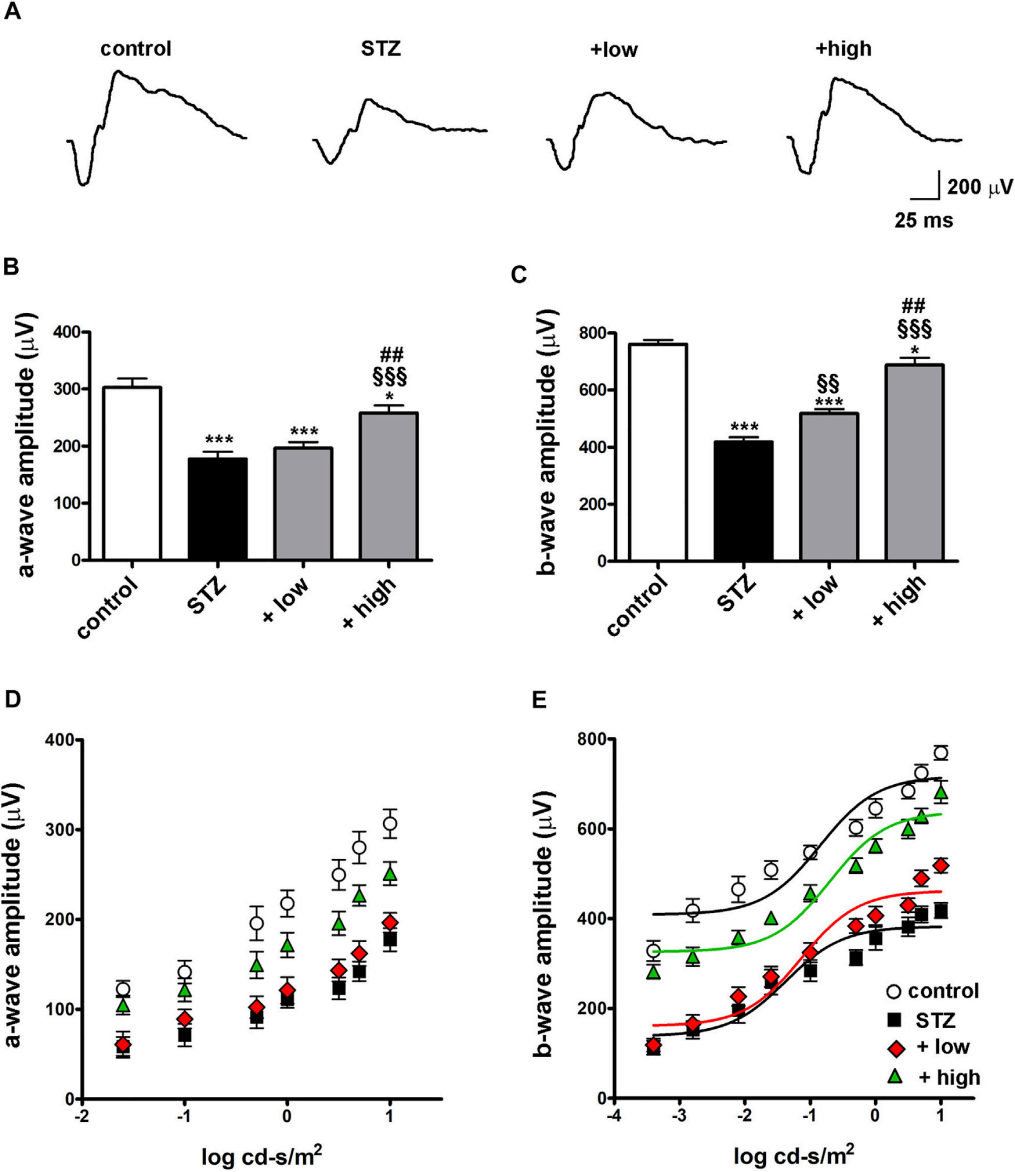
Effects of the compound on scotopic ERG responses **(A)** Representative scotopic ERG waveforms recorded at one log cd-s/m^2^ light intensity. Quantitative analysis of the scotopic a- **(B)** and b-wave **(C)** amplitudes recorded at one log cd-s/m^2^ light intensity in controls, STZ-untreated rats or STZ rats treated with either the low dose (C3G, 1.0 mg; verbascoside, 0.25 mg and zinc, 0.125 mg) or the high dose (C3G, 3.0 mg; verbascoside, 0.75 mg and zinc, 0.375 mg) of the compound. Statistical significance was assessed by one-way ANOVA followed by Tukey’s multiple comparison post-hoc test (N = 8). ^*^ *p* < 0.05 and ^***^ *p* < 0.001 vs control; ^§§^ *p* < 0.01 and ^§§§^ *p* < 0.001 vs STZ; ^##^ *p* < 0.01 vs low dose treated STZ. Quantitative analysis of the scotopic a- **(D)** and b-wave **(E)** amplitudes recorded at increasing light intensities. Lines indicate the Naka-Rushton fit of the variation of the b-wave amplitude depending on stimulus intensity. Statistical significance was assessed by two-ways ANOVA followed by Bonferroni’s multiple comparison post-hoc test (N = 8).

To evaluate whether protective efficacy of the compound might be reflected on preventing STZ-induced visual dysfunction, we analyzed the outer and inner retinal activity using scotopic ERG recordings. Under scotopic condition, a-wave reflects the activity of rods, while b-wave reflects the activity of bipolar cells and Müller glia. Figure 7A shows representative mixed a- and b-waves recorded at light intensities of one log cd-s/m^2^. As shown in Figures 7B, C, In STZ rats treated with the low dose, ERG responses were partially preserved. In fact, at maximal stimulus intensity of 1 log cd-s/m^2^, the a-wave amplitude, although not significantly different from that measured in STZ rats (196.5 ± 10.8, *p* > 0.05 vs STZ), showed a tendency toward an increase, while the b-wave amplitude was significantly higher than in STZ rats (518.2 ± 15.7, *p* < 0.01 vs STZ). After treatment with the high dose, a-and b-wave amplitudes were significantly higher than in STZ rats (a-wave 251.2 ± 12.8; b-wave 682.2 ± 24.8 *p* < 0.001 vs STZ) and in low dose-treated rats (*p* < 0.01 vs low dose), but still lower than in controls (*p* < 0.05). No significant effects on ERG responses were found in control rats treated with the compound either at low or high dose (Supplementary Figure S2). As shown in Figures 7D, E, a- and b-wave amplitude increased with increasing stimulus intensity. A clear a-wave developed at a light intensity of approximately −1.6 log cd-s/m^2^. Compared to controls, STZ rats showed a reduction in the amplitude of both the a-wave and the b-wave at light intensities ranging from −1.6 to one log cd-s/m^2^ (*p* < 0.001 vs control) although the thickness of both ONL and INL measured in untreated STZ rats did not differ from that measured in controls (Supplementary Figure S3). As shown in Figure 7E, b-wave amplitudes over increasing light intensities were fitted using the Naka-Rushton equation to evaluate the post-receptor response amplitude (Vmax) and the retinal sensitivity (k). As shown in Table 2, in STZ rats, the values of Vmax and k were significantly lower than in controls (*p* < 0.001). In STZ rats treated with the low dose, Vmax values were significantly higher than those in untreated STZ rats, whereas k values were almost comparable. On the contrary, in STZ rats treated with the high dose, the values of both Vmax and k were significantly higher than in untreated STZ rats.

**TABLE 2:** Parameters obtained from b-wave amplitude using the Naka-Rushton function.

	Control	STZ	+ Low	+ High
Vmax (µV)	717.5 ± 16.5	382.9 ± 11.7^***^	462.4 ± 12.5^***,§^	638.7 ± 14.2^**,^§§^,##^
k (log cd-s/m^2^)	−0.87 ± 0.12	−1.40 ± 0.11^**^	−1.31 ± 0.12^*^	−0.75 ± 0.11§§^,#^

*p < 0.05, **p < 0.0, ***p < 0.001 vs control, ^§^p < 0.01, ^§§^p < 0.001 vs STZ, ^#^p < 0.01, ^##^p < 0.001 vs low dose.

## Discussion

Recently, much effort has been devoted to preclinical studies in which preventive treatments have been tested in order to stop/delay DR progression. Here, preclinical data on the efficacy of antioxidant compounds in preventing the occurrence of pathological signs that characterize DR have been collected and discussed in light of their possible application to humans.

Currently, the recommended treatment for severe non-proliferative or proliferative DR is photocoagulation and intravitreal injections of anti-VEGF associated, or not, with focal laser for diabetic macular oedema (DME). Anti-VEGF therapy not only counteracts new vessel proliferation but also interferes with inflammatory processes that have a considerable role in the pathogenesis of DME. In particular, VEGF accumulation induced by high blood glucose triggers major inflammatory processes and several drugs are already approved in clinical practice to handle DME. Anti-VEGF agents act also with mechanisms that interfere with inflammatory pathways. In particular, inflammation-induced dropout of pericytes leads to the formation of aberrant capillaries through VEGF accumulation, which reverberates on inflammatory pathways thus contributing to further compromise pericyte viability (Giurdanella et al., 2015). The complex network of pro-inflammatory factors involved in DME represents the rationale for its treatment by intravitreal steroids. In particular, repeated dexamethasone implants have been found to correlate well with DME duration with a better efficacy than anti-VEGF treatments (Bucolo et al., 2018).

Among the major limitations of treatments against vascular complications that characterize DR, late beginning of therapy is the greatest obstacle to cure the disease. In fact, in a high percentage of diabetic patients, inadequate control of metabolic parameters is a major cause of chronic complications including progressive DR. Approximately 15% of patients may show some degree of DR at the time of diabetes first diagnosis although severe microvascular complications will develop many years later. Early diagnosis of DR is the best tool to prevent or delay vision loss, but DR has been long considered as an asymptomatic disease and its progression to advanced stages has reduced the effectiveness of treatments (Corcóstegui et al., 2017; Rodríguez et al., 2019). Current work using subtle analysis of ERG waveforms in diabetic patients has allowed ophthalmologists to reach early diagnosis of non-proliferative DR and to evaluate the possibility to intervene at early stages when the retinal neuro-vascular unit is not yet seriously compromised (Ahmadieh et al., 2021). In this respect, natural plant extracts or their naturally occurring components have been shown to be very proficient in the prevention and treatment of DR. In preclinical studies, treatments with protective compounds that inhibit oxidative stress and inflammation have been shown to counteract the pathological signs of DR although clinical trials dealing with the effect of antioxidants on human DR have provided limited results that are often controversial (Garcia-Medina et al., 2020).

At the preclinical level, the STZ model of diabetes is a well-established and useful tool to investigate complications of diabetes including DR, although its progression to visual dysfunction, which in humans take years to be established, in rodents occur early after STZ injection. However, some limitations need to be considered when approaching the translation to clinics. The induction of diabetes with STZ consists in the acute disruption of pancreatic beta cells and the drastic interruption of insulin production. Therefore, it results in an all-or-nothing phenomenon in which different predispositions or risks of diabetes and, subsequently, different risks of developing DR, are not reproducible.

After STZ injection, elevated blood glucose concentration induced by pancreatic β-cell disruption, initiates an early increase of ROS, which overwhelms endogenous antioxidant defences and leads to early inflammation, increased levels of both HIF-1α and VEGF, increased vascular permeability that causes BRB breakdown leading to decreased visual function (Rodríguez et al., 2019).

There is convincing evidence that throughout the pathologic process of DR, oxidative stress plays an important role. In fact, a series of metabolic pathways altered by the exposure to high glucose concurrently result in the increment of ROS production in the retina. The excess in glucose undergoing glycolysis and citric acid cycle compels the increment in mitochondrial electron transport reaching the maximum threshold, and thus forcing the electron transfer to molecular oxygen generating radical species. In addition, chronic exposure to hyperglycemia favors the nonenzymatic glycation of proteins and lipids leading to the accumulation of advanced glycation end products (AGEs). Besides causing the loss of protein structure and function, AGEs can bind their receptors driving the downstream activation of NADPH oxidases, thus enhancing ROS generation. ROS accumulation triggers the endogenous antioxidant response masterly regulated by the redox sensitive transcriptional factor NRF2, which in turn mediates the increment in antioxidant enzymes such as HO-1 (Kang and Yang, 2020). However, the high glucose-driven ROS accumulation overcomes the endogenous capacity of antioxidant enzymes, which is further hindered by the high-glucose mediated activation of metabolic pathways driving the depletion of enzymatic cofactors. Both the ROS increment and the antioxidant depotentiation initiate a cascade of events leading to retinal damage (Kowluru and Chan, 2007).

ROS increase also reverberates on the stimulation of the inflammatory response that further promotes oxidative damage. Indeed, ROS accumulation triggers the activation of NF-kB, a transcription factor involved in the modulation of the inflammatory response, which in turn regulates the expression of pro-inflammatory cytokines, such as IL-6 (Liu et al., 2017). In particular, inflammatory cytokines contribute to further amplify retinal cell damage by recruiting and activating immune cells as well as by increasing the levels of mediators responsible for the BRB dysfunction (Semeraro et al., 2015).

Pro-inflammatory stimuli result in macroglia activation as identified by GFAP overexpression, which can be considered as a marker for gliosis. In the healthy retina, GFAP is expressed only in astrocytes and not in Müller cells, whereas in the diseased retina, Müller cells exhibit dense GFAP immunostaining that has been widely used as a cellular marker for retinal pathological injury (Vecino et al., 2016).

Inflammation and gliosis lead to cell injury both in the neuroretina and in retinal blood vessels, thus promoting neuronal and vascular dysfunction. In particular, capillary closure and non-perfusion that develop relatively early after the onset of diabetes participate to establish hypoxic condition in the retina. Hypoxia causes the upregulation of HIF-1α, although HIF-1α increase may also occur as a direct consequence of hyperglycaemia independently on hypoxia (Xiao et al., 2013). HIF-1α accumulation leads to an increased production of VEGF that is a target of HIF-1. Upregulation of the VEGF signalling pathway ultimately leads to the dysfunction of inner and outer BRB with the loss of tight junctions integrity between neighbouring endothelial cells of both retinal microvasculature endothelium and RPE cells (Antonetti et al., 1999). Tight junction proteins including Claudin five and ZO-1, play a pivotal role in maintaining the BRB function through the regulation of the transport of solutes and molecules (Eshaq et al., 2017). The BRB breakdown results in the leakage of blood contents from retinal and choroidal vessels to the surrounding tissue which reverberate on inflammatory and ischemic processes leading to neuroretinal cell damage and ERG dysfunction (Xu and Le, 2011; Klaassen et al., 2013).

Retinal cell death is driven by the activation of apoptotic cascade, as also confirmed here by the altered Bax/Bcl-2 ratio, ultimately leading to the activation of caspase 3, a critical enzyme involved in apoptosis execution. As shown by the present findings, active caspase 3 is mostly localized to the inner retina in agreement with previous results indicating a significant involvement of inner retinal cells in apoptotic processes that characterize DR (Li et al., 2008; Sasaki et al., 2010; Thounaojam et al., 2017; Amato et al., 2018) while the outer retina is rather preserved at least soon after blood glucose increase (Park et al., 2003).

As a consequence of retinal damage, ERG becomes dysfunctional as demonstrated by the decreased amplitude of the scotopic a-wave arising from photoreceptor hyperpolarization, and the b-wave that reflects bipolar cell depolarization leading to potassium outflux that is buffered by Muller cells, which produce a transretinal current participating to b-wave generation (Dong and Hare, 2000 PMID 10824262). ERG dysfunction occurs at 4 weeks after STZ injection in agreement with previous longitudinal studies demonstrating that in the STZ model, ERG responses are unaffected by hyperglycemia up to 3 weeks after diabetes onset (Li et al., 2002; Shinoda et al., 2007; Cammalleri et al., 2017; Amato et al., 2018).

Impairment of scotopic ERG components might be reconducted to alterations of the activity of both photoreceptors and inner retinal cells. In this respect, despite no evidence of reduced thickness of the outer retina in which no apoptotic activity was detected, decreased amplitude of the a-wave is indicative of early photoreceptor suffering in line with previous reports demonstrating early degenerative changes of photoreceptors and pigment epithelium prior to apoptotic events (Énzsöly et al., 2014; Liu et al., 2016). On the other hand, the increased apoptotic activity in the inner retina as demonstrated by the presence of caspase 3-positive cells together with Müller cell gliosis, correlates well with altered b-waves that are known to be early and extensively affected in DR as a sign of inner retina dysfunction (Li et al., 2002).

Ongoing apoptotic processes in the inner retina were not correlated with reduced thickness of the INL in line with previous findings demonstrating that apoptosis becomes manifest as early as 4 weeks after STZ injection to then progress over an extended period of time, eventually leading to a later reduction in the retinal layer thickness (Barber et al., 1998).

As shown by the present results hyperglycaemia-induced cascade leading to ERG dysfunction is prevented by diet administration with antioxidant compounds including C3G, verbascoside and zinc starting on the day of STZ injection and persisting until the fourth week after, when the rats have undergone to ERG recording.

Most chronic diseases including diabetes are worsened by a deficiency of essential antioxidant nutrients, a condition that is further impaired by deficits in their absorption and utilization (Shi et al., 2020). Although major efforts to support the clinical benefits of vitamin and antioxidant interventions to reduce the risk and severity of vision loss, nutraceutical therapy is still limited and more large scales studies in DR are needed to overcome the limitations of clinical trials. Large clinical trials such as the AREDS2 formulation was a major improvement, bringing together natural antioxidants for the treatment of macular degeneration (AREDS2 Research Group et al., 2012; Shi et al., 2020). In this respect, decreased incidence of blindness in patients with macular degeneration have been observed in the zinc supplemented elderly (Prasad and Bao, 2019). Results from clinical trials about the efficacy of natural antioxidants in DR patients are highly variable and, in some cases, controversial. However, a general tendency towards the efficacy of antioxidants in combination has been reported to match the need of counteracting the wide spectrum of pathogenic mechanisms characterizing DR (Garcia-Medina et al., 2020). For instance, the supplementation with lutein, alpha-tocopherol, niacin, beta-carotene, zinc and selenium has been found to delay DR progression in patients with type 2 diabetes, although no effects on visual acuity have been detected (Garcia-Medina et al., 2011). In contrast, ameliorated visual acuity, but no efficacy on retinal thickness have been determined in patients with type 2 diabetes following the treatment with combined antioxidants including carotenoids, racemic compounds, vitamins and botanical extracts (Chous et al., 2016).

Still, major limitation to the conflicting results of clinical trials in DR depends on the fact that antioxidant efficacy of natural compounds is generally tested on patients who already experienced disorders of visual processing at various levels. Instead, the specific interest would be to test the antioxidant efficacy of nutraceutical compounds by intervening on the early stages of DR to prevent/delay its progression to proliferative DR. In this respect, there is an increasing body of evidence about the role of antioxidants in the control of DR in animal models.

The present results in the STZ model of DR demonstrate the preventive efficacy of a novel compound containing C3G, verbascoside and zinc as nutrients with antioxidant and anti-inflammatory properties. The PK profile of C3G has been characterized by de Ferrars et al. (2014) demonstrating C3G retention in the plasma up to 6 h post-oral administration. In a recent work, C3G has been detected in the plasma from which it reaches the ocular tissue following oral administration (Amato et al., 2021) also in line with previous findings (Matsumoto et al., 2006). Orally administered verbascoside appears to be distributed to most tissues including the brain suggesting its ability to cross the blood-brain barrier (Wen et al., 2016). In addition, verbascoside, has been found to protect ocular tissues and fluids from naturally occurring oxidation (Mosca et al., 2014). The PK profile and biodistribution of zinc have been established in previous studies (Nève et al., 1991; Gilbert et al., 2019). Recently, zinc, orally administered, has been found to accumulate in the retina where it acts as cofactor for antioxidant enzymes (Kamińska et al., 2021). C3G is a potent inhibitor of oxidative stress and its antioxidant properties include the activation of endogenous anti-oxidant enzymes, quenching singlet oxygen, chelation of trace metals involved in free radical production, inhibition of ROS-promoting enzymes, ROS scavenger properties (Tena et al., 2020). ROS scavenging activates a downstream anti-inflammatory cascade leading to the inhibition of the NF-kB activity, the reduced release of pro-inflammatory cytokines and the inhibition of inflammasome activity (Min et al., 2010; Jin et al., 2018). Additional efficacy of C3G includes its capacity to counteract pathological neoangiogenesis by contrasting VEGF overexpression in response to high glucose (Matsunaga et al., 2010; Oliveira et al., 2020). Verbascoside has been reported to exert a major anti-inflammatory activity through the inhibition of NF-kB signalling and nitric oxide pathway, thus resulting in decreased inflammatory response (Wu et al., 2020). Interestingly, verbascoside has also been shown to directly interact with cell survival mechanisms by inhibiting autophagy-induced apoptosis (Chen et al., 2019). In diabetic condition, verbascoside has been established to inhibit endoplasmic reticulum stress and advanced glycation end-product formation (Liu et al., 2013; Galli et al., 2020). Zinc is known as a fundamental trace element involved in the structure and function of numerous enzymes regulating cellular processes and signalling pathways. Zinc potentiates the endogenous antioxidant response by increasing the activity of antioxidant proteins and enzymes that are transcribed by NRF2 (Jarosz et al., 2017). Zinc deficiency has been shown to correlate with ocular abnormalities such as cataract and retinal diseases including age-related macular degeneration and DR (Miao et al., 2013). In line with the negative effects of zinc deficiency, the supplementation of zinc, in combination with additional functional nutrients, has provided promising results to establish its potential for counteracting retinal diseases (Miao et al., 2013; Vishwanathan et al., 2013). However, the boundary line between the activity and the contribution of these hypothetical classes of natural molecules is complicated by the fact that many of the properties of each molecule are shared with the others. For instance, C3G may also activate endogenous antioxidant defenses, as played by zinc. In addition, verbascoside may exert an anti-inflammatory activity leading to reduced ROS generation thus amplifying the effect of zinc and C3G. In this respect, although a theoretical classification of the molecules could be done based on their individual bioactivity, an actual discrimination of each component activity in the context of a compound is difficult to retrieve. The efficacy of C3G, verbascoside and zinc has already been established in a model of light induced retinal damage in which the combined formula has been shown to exert marked antioxidant and anti-inflammatory effects resulting in a significant protection of photoreceptor morpho-functional integrity (Amato et al., 2021). Its application to DR is further stressed by the hypoglycaemic properties of the compounds included in the formula (Sasaki et al., 2007; Xiong et al., 2013; Sadri et al., 2017). However, as shown by the present results, glycemia is not affected by the formula presumably because the serious destruction of islet function by STZ renders the blood glucose difficult to control (Pang et al., 2020). Although the efficacy of the compound supplementation on pathological signs of DR is independent on glycemia control, oxidative stress- and inflammation-related mechanisms downstream hyperglycaemia are markedly counteracted with a significant counterpart in preventing BRB leakage, retinal cell death and retinal dysfunction.

## Conclusion

One the major clinical problems of late stages of DR is irreversible visual loss due to the scarce availability of drugs restoring visual function once proliferative DR is established. Therefore, research work to investigate possible strategies to prevent DR progression should be accurately pursued. The fact that antioxidant/anti-inflammatory compounds, possibly through their protective efficacy on vascular damage may prevent the apoptotic cascade leading to ERG dysfunction, adds further relevance to their potential application as a preventive therapy to counteract DR progression. On the other hand, the glycemic control is the milestone for the management of diabetes and its related complications including DR. However, a significant number of diabetic patients undergoing glycemic control therapies still develop DR symptoms, thus highlighting the need of complementary therapies, other than those dedicated to blood glucose lowering. The present study demonstrates that antioxidant supplementation could represent a non-invasive solution to prevent or delay DR signs. Therefore, the translational goal of the present approach consists in its potential complementary role in combination with hypoglycemic drugs in order to further reduce the risk of DR onset and progression. However, the extrapolation of these experimental findings to the clinic is not straightforward as animal models of DR may not faithfully recapitulate all the pathologic signs seen in human DR. In this respect, neither macular edema nor proliferative retinopathy ever develop in STZ-rats, indicating that they are a suitable model for the early phase of human DR.

## Data Availability

The raw data supporting the conclusions of this article will be made available by the authors, without undue reservation.

## References

[B1] Ahmadieh H. Behbahani S. Safi S. (2021). Continuous Wavelet Transform Analysis of ERG in Patients with Diabetic Retinopathy. Doc. Ophthalmol. 142 (3), 305–314. 10.1007/s10633-020-09805-9 33226538

[B2] Al-Kharashi A. S. (2018). Role of Oxidative Stress, Inflammation, Hypoxia and Angiogenesis in the Development of Diabetic Retinopathy. Saudi J. Ophthalmol. 32 (4), 318–323. 10.1016/j.sjopt.2018.05.002 30581303 PMC6300752

[B3] Alipieva K. Korkina L. Orhan I. E. Georgiev M. I. (2014). Verbascoside--a Review of its Occurrence, (Bio)synthesis and Pharmacological Significance. Biotechnol. Adv. 32 (6), 1065–1076. 10.1016/j.biotechadv.2014.07.001 25048704

[B4] Amato R. Canovai A. Melecchi A. Pezzino S. Corsaro R. Dal Monte M. (2021). Dietary Supplementation of Antioxidant Compounds Prevents Light-Induced Retinal Damage in a Rat Model. Biomedicines 9 (9), 1177. 10.3390/biomedicines9091177 34572363 PMC8472009

[B5] Amato R. Rossino M. G. Cammalleri M. Locri F. Pucci L. Dal Monte M. (2018). Lisosan G Protects the Retina from Neurovascular Damage in Experimental Diabetic Retinopathy. Nutrients 10 (12), 1932. 10.3390/nu10121932 PMC6316708 30563182

[B6] Antonetti D. A. Barber A. J. Hollinger L. A. Wolpert E. B. Gardner T. W. (1999). Vascular Endothelial Growth Factor Induces Rapid Phosphorylation of Tight junction Proteins Occludin and Zonula Occluden 1. A Potential Mechanism for Vascular Permeability in Diabetic Retinopathy and Tumors. J. Biol. Chem. 274 (33), 23463–23467. 10.1074/jbc.274.33.23463 10438525

[B7] AREDS2 Research Group Chew E. Y. Chew E. Y. Clemons T. SanGiovanni J. P. Danis R. (2012). The Age-Related Eye Disease Study 2 (AREDS2): Study Design and Baseline Characteristics (AREDS2 Report Number 1). Ophthalmology 119 (11), 2282–2289. 10.1016/j.ophtha.2012.05.027 22840421 PMC3485447

[B8] Barber A. J. Baccouche B. (2017). Neurodegeneration in Diabetic Retinopathy: Potential for Novel Therapies. Vis. Res. 139, 82–92. 10.1016/j.visres.2017.06.014 28988945

[B9] Barber A. J. Lieth E. Khin S. A. Antonetti D. A. Buchanan A. G. Gardner T. W. (1998). Neural Apoptosis in the Retina during Experimental and Human Diabetes. Early Onset and Effect of Insulin. J. Clin. Invest. 102 (4), 783–791. 10.1172/JCI2425 9710447 PMC508941

[B11] Bucolo C. Gozzo L. Longo L. Mansueto S. Vitale D. C. Drago F. (2018). Long-term Efficacy and Safety Profile of Multiple Injections of Intravitreal Dexamethasone Implant to Manage Diabetic Macular Edema: A Systematic Review of Real-World Studies. J. Pharmacol. Sci. 138 (4), 219–232. 10.1016/j.jphs.2018.11.001 30503676

[B12] Burgos C. Muñoz-Mingarro D. Navarro I. Martín-Cordero C. Acero N. (2020). Neuroprotective Potential of Verbascoside Isolated from Acanthus Mollis L. Leaves through its Enzymatic Inhibition and Free Radical Scavenging Ability. Antioxidants (Basel) 9 (12), 1207. 10.3390/antiox9121207 PMC7759776 33266151

[B13] Cammalleri M. Locri F. Marsili S. Dal Monte M. Pisano C. Mancinelli A. (2017). The Urokinase Receptor-Derived Peptide UPARANT Recovers Dysfunctional Electroretinogram and Blood-Retinal Barrier Leakage in a Rat Model of Diabetes. Invest. Ophthalmol. Vis. Sci. 58 (7), 3138–3148. 10.1167/iovs.17-21593 28632880

[B14] Chen Q. Xi X. Zeng Y. He Z. Zhao J. Li Y. (2019). Acteoside Inhibits Autophagic Apoptosis of Retinal Ganglion Cells to rescue Glaucoma-Induced Optic Atrophy. J. Cel Biochem. 120 (8), 13133–13140. 10.1002/jcb.28586 PMC6618276 31021425

[B15] Chous A. P. Richer S. P. Gerson J. D. Kowluru R. A. (2016). The Diabetes Visual Function Supplement Study (DiVFuSS). Br. J. Ophthalmol. 100 (2), 227–234. 10.1136/bjophthalmol-2014-306534 26089210 PMC4752618

[B16] Corcóstegui B. Durán S. González-Albarrán M. O. Hernández C. Ruiz-Moreno J. M. Salvador J. (2017). Update on Diagnosis and Treatment of Diabetic Retinopathy: A Consensus Guideline of the Working Group of Ocular Health (Spanish Society of Diabetes and Spanish Vitreous and Retina Society). J. Ophthalmol. 2017, 8234186. 10.1155/2017/8234186 28695003 PMC5488240

[B17] de Ferrars R. M. Czank C. Zhang Q. Botting N. P. Kroon P. A. Cassidy A. (2014). The Pharmacokinetics of Anthocyanins and Their Metabolites in Humans. Br. J. Pharmacol. 171 (13), 3268–3282. 10.1111/bph.12676 24602005 PMC4080980

[B18] Dong C. J. Hare W. A. (2000). Contribution to the Kinetics and Amplitude of the Electroretinogram B-Wave by Third-Order Retinal Neurons in the Rabbit Retina. Vis. Res. 40 (6), 579–589. 10.1016/s0042-6989(99)00203-5 10824262

[B19] Énzsöly A. Szabó A. Kántor O. Dávid C. Szalay P. Szabó K. (2014). Pathologic Alterations of the Outer Retina in Streptozotocin-Induced Diabetes. Invest. Ophthalmol. Vis. Sci. 55 (6), 3686–3699. 10.1167/iovs.13-13562 24845643

[B20] Eshaq R. S. Aldalati A. M. Z. Alexander J. S. Harris N. R. (2017). Diabetic Retinopathy: Breaking the Barrier. Pathophysiology 24 (4), 229–241. 10.1016/j.pathophys.2017.07.001 28732591 PMC5711541

[B21] Feng Y. Busch S. Gretz N. Hoffmann S. Hammes H. P. (2012). Crosstalk in the Retinal Neurovascular Unit - Lessons for the Diabetic Retina. Exp. Clin. Endocrinol. Diabetes 120 (4), 199–201. 10.1055/s-0032-1304571 22402950

[B22] Furman B. L. (2015). Streptozotocin-Induced Diabetic Models in Mice and Rats. Curr. Protoc. Pharmacol. 70, 5.47.1–5.47.20. 10.1002/0471141755.ph0547s70 26331889

[B23] Galli A. Marciani P. Marku A. Ghislanzoni S. Bertuzzi F. Rossi R. (2020). Verbascoside Protects Pancreatic β-Cells against ER-Stress. Biomedicines 8 (12), 582. 10.3390/biomedicines8120582 PMC7762434 33302345

[B24] Garcia-Medina J. J. Pinazo-Duran M. D. Garcia-Medina M. Zanon-Moreno V. Pons-Vazquez S. (2011). A 5-year Follow-Up of Antioxidant Supplementation in Type 2 Diabetic Retinopathy. Eur. J. Ophthalmol. 21 (5), 637–643. 10.5301/EJO.2010.6212 21218388

[B25] Garcia-Medina J. J. Rubio-Velazquez E. Foulquie-Moreno E. Casaroli-Marano R. P. Pinazo-Duran M. D. Zanon-Moreno V. (2020). Update on the Effects of Antioxidants on Diabetic Retinopathy: *In Vitro* Experiments, Animal Studies and Clinical Trials. Antioxidants (Basel) 9 (6), 561. 10.3390/antiox9060561 PMC7346101 32604941

[B26] Gilbert R. Peto T. Lengyel I. Emri E. (2019). Zinc Nutrition and Inflammation in the Aging Retina. Mol. Nutr. Food Res. 63 (15), e1801049. 10.1002/mnfr.201801049 31148351

[B27] Giurdanella G. Anfuso C. D. Olivieri M. Lupo G. Caporarello N. Eandi C. M. (2015). Aflibercept, Bevacizumab and Ranibizumab Prevent Glucose-Induced Damage in Human Retinal Pericytes *In Vitro*, through a PLA2/COX-2/VEGF-A Pathway. Biochem. Pharmacol. 96 (3), 278–287. 10.1016/j.bcp.2015.05.017 26056075

[B28] Gu L. Xu H. Zhang C. Yang Q. Zhang L. Zhang J. (2019). Time-dependent Changes in Hypoxia- and Gliosis-Related Factors in Experimental Diabetic Retinopathy. Eye (Lond) 33 (4), 600–609. 10.1038/s41433-018-0268-z 30401898 PMC6461831

[B29] Jarosz M. Olbert M. Wyszogrodzka G. Młyniec K. Librowski T. (2017). Antioxidant and Anti-inflammatory Effects of Zinc. Zinc-dependent NF-κB Signaling. Inflammopharmacology 25 (1), 11–24. 10.1007/s10787-017-0309-4 28083748 PMC5306179

[B30] Jin X. Wang C. Wu W. Liu T. Ji B. Zhou F. (2018). Cyanidin-3-glucoside Alleviates 4-Hydroxyhexenal-Induced NLRP3 Inflammasome Activation via JNK-C-Jun/AP-1 Pathway in Human Retinal Pigment Epithelial Cells. J. Immunol. Res. 2018, 5604610. 10.1155/2018/5604610 29854843 PMC5952446

[B31] Kamińska A. Romano G. L. Rejdak R. Zweifel S. Fiedorowicz M. Rejdak M. (2021). Influence of Trace Elements on Neurodegenerative Diseases of the Eye-The Glaucoma Model. Int. J. Mol. Sci. 22 (9), 4323. 10.3390/ijms22094323 33919241 PMC8122456

[B32] Kang Q. Yang C. (2020). Oxidative Stress and Diabetic Retinopathy: Molecular Mechanisms, Pathogenetic Role and Therapeutic Implications. Redox Biol. 37, 101799. 10.1016/j.redox.2020.101799 33248932 PMC7767789

[B33] Khoo H. E. Azlan A. Tang S. T. Lim S. M. (2017). Anthocyanidins and Anthocyanins: Colored Pigments as Food, Pharmaceutical Ingredients, and the Potential Health Benefits. Food Nutr. Res. 61 (1), 1361779. 10.1080/16546628.2017.1361779 28970777 PMC5613902

[B34] Klaassen I. Van Noorden C. J. Schlingemann R. O. (2013). Molecular Basis of the Inner Blood-Retinal Barrier and its Breakdown in Diabetic Macular Edema and Other Pathological Conditions. Prog. Retin. Eye Res. 34, 19–48. 10.1016/j.preteyeres.2013.02.001 23416119

[B35] Kowluru R. A. Chan P. S. (2007). Oxidative Stress and Diabetic Retinopathy. Exp. Diabetes Res. 2007, 43603. 10.1155/2007/43603 17641741 PMC1880867

[B36] Leena M. M. Silvia M. G. Vinitha K. Moses J. A. Anandharamakrishnan C. (2020). Synergistic Potential of Nutraceuticals: Mechanisms and Prospects for Futuristic Medicine. Food Funct. 11 (11), 9317–9337. 10.1039/d0fo02041a 33211054

[B37] Li Q. Zemel E. Miller B. Perlman I. (2002). Early Retinal Damage in Experimental Diabetes: Electroretinographical and Morphological Observations. Exp. Eye Res. 74 (5), 615–625. 10.1006/exer.2002.1170 12076083

[B38] Li W. Chen S. Zhou G. Li H. Zhong L. Liu S. (2018). Potential Role of Cyanidin 3-glucoside (C3G) in Diabetic Cardiomyopathy in Diabetic Rats: An *In Vivo* Approach. Saudi J. Biol. Sci. 25 (3), 500–506. 10.1016/j.sjbs.2016.11.007 29686513 PMC5910639

[B39] Li Y. H. Zhuo Y. H. Lü L. Chen L. Y. Huang X. H. Zhang J. L. (2008). Caspase-dependent Retinal Ganglion Cell Apoptosis in the Rat Model of Acute Diabetes. Chin. Med. J. (Engl) 121 (24), 2566–2571. 10.1097/00029330-200812020-00018 19187597

[B40] Liu H. Tang J. Du Y. Saadane A. Tonade D. Samuels I. (2016). Photoreceptor Cells Influence Retinal Vascular Degeneration in Mouse Models of Retinal Degeneration and Diabetes. Invest. Ophthalmol. Vis. Sci. 57 (10), 4272–4281. 10.1167/iovs.16-19415 27548901 PMC5015983

[B41] Liu T. Zhang L. Joo D. Sun S. C. (2017). NF-κB Signaling in Inflammation. Signal. Transduct. Target. Ther. 2, 17023. 10.1038/sigtrans.2017.23 29158945 PMC5661633

[B42] Liu Y. H. Lu Y. L. Han C. H. Hou W. C. (2013). Inhibitory Activities of Acteoside, Isoacteoside, and its Structural Constituents against Protein Glycation *In Vitro* . Bot. Stud. 54 (1), 6. 10.1186/1999-3110-54-6 28510849 PMC5432847

[B43] Marreiro D. D. Cruz K. J. Morais J. B. Beserra J. B. Severo J. S. de Oliveira A. R. (2017). Zinc and Oxidative Stress: Current Mechanisms. Antioxidants (Basel) 6 (2), 24. 10.3390/antiox6020024 PMC5488004 28353636

[B44] Matsumoto H. Nakamura Y. Iida H. Ito K. Ohguro H. (2006). Comparative Assessment of Distribution of Blackcurrant Anthocyanins in Rabbit and Rat Ocular Tissues. Exp. Eye Res. 83 (2), 348–356. 10.1016/j.exer.2005.12.019 16635490

[B45] Matsunaga N. Tsuruma K. Shimazawa M. Yokota S. Hara H. (2010). Inhibitory Actions of Bilberry Anthocyanidins on Angiogenesis. Phytother Res. 24 (Suppl. 1), S42–S47. 10.1002/ptr.2895 19496063

[B46] Mi X. S. Yuan T. F. Ding Y. Zhong J. X. So K. F. (2014). Choosing Preclinical Study Models of Diabetic Retinopathy: Key Problems for Consideration. Drug Des. Devel. Ther. 8, 2311–2319. 10.2147/DDDT.S72797 PMC4242133 25429204

[B47] Miao X. Sun W. Miao L. Fu Y. Wang Y. Su G. (2013). Zinc and Diabetic Retinopathy. J. Diabetes Res. 2013, 425854. 10.1155/2013/425854 23671870 PMC3647550

[B48] Min S. W. Ryu S. N. Kim D. H. (2010). Anti-inflammatory Effects of Black rice, Cyanidin-3-O-Beta-D-Glycoside, and its Metabolites, Cyanidin and Protocatechuic Acid. Int. Immunopharmacol. 10 (8), 959–966. 10.1016/j.intimp.2010.05.009 20669401

[B49] Mosca M. Ambrosone L. Semeraro F. Casamassima D. Vizzarri F. Costagliola C. (2014). Ocular Tissues and Fluids Oxidative Stress in Hares Fed on Verbascoside Supplement. Int. J. Food Sci. Nutr. 65 (2), 235–240. 10.3109/09637486.2013.836742 24059688

[B50] Naderi A. Zahed R. Aghajanpour L. Amoli F. A. Lashay A. (2019). Long Term Features of Diabetic Retinopathy in Streptozotocin-Induced Diabetic Wistar Rats. Exp. Eye Res. 184, 213–220. 10.1016/j.exer.2019.04.025 31028750

[B51] Nair A. B. Jacob S. (2016). A Simple Practice Guide for Dose Conversion between Animals and Human. J. Basic Clin. Pharm. 7 (2), 27–31. 10.4103/0976-0105.177703 27057123 PMC4804402

[B52] Naka K. I. Rushton W. A. (1966). S-potentials from Colour Units in the Retina of Fish (Cyprinidae). J. Physiol. 185 (3), 536–555. 10.1113/jphysiol.1966.sp008001 5918058 PMC1395833

[B53] Nève J. Hanocq M. Peretz A. Abi Khalil F. Pelen F. Famaey J. P. (1991). Pharmacokinetic Study of Orally Administered Zinc in Humans: Evidence for an Enteral Recirculation. Eur. J. Drug Metab. Pharmacokinet. 16 (4), 315–323. 10.1007/BF03189977 1823876

[B54] Nomi Y. Iwasaki-Kurashige K. Matsumoto H. (2019). Therapeutic Effects of Anthocyanins for Vision and Eye Health. Molecules 24 (18), 3311. 10.3390/molecules24183311 PMC6767261 31514422

[B55] Oliveira H. Fernandes A. F Brás N. Mateus N. de Freitas V. Fernandes I. (2020). Anthocyanins as Antidiabetic Agents-In Vitro and In Silico Approaches of Preventive and Therapeutic Effects. Molecules 25 (17), 3813. 10.3390/molecules25173813 PMC7504281 32825758

[B56] Pang B. Ni Q. Di S. Du L. J. Qin Y. L. Li Q. W. (2020). Luo Tong Formula Alleviates Diabetic Retinopathy in Rats through Micro-200b Target. Front. Pharmacol. 11, 551766. 10.3389/fphar.2020.551766 33324202 PMC7723456

[B57] Park S. H. Park J. W. Park S. J. Kim K. Y. Chung J. W. Chun M. H. (2003). Apoptotic Death of Photoreceptors in the Streptozotocin-Induced Diabetic Rat Retina. Diabetologia 46 (9), 1260–1268. 10.1007/s00125-003-1177-6 12898017

[B58] Perron N. R. Brumaghim J. L. (2009). A Review of the Antioxidant Mechanisms of Polyphenol Compounds Related to Iron Binding. Cell Biochem. Biophys. 53 (2), 75–100. 10.1007/s12013-009-9043-x 19184542

[B59] Prasad A. S. Bao B. (2019). Molecular Mechanisms of Zinc as a Pro-antioxidant Mediator: Clinical Therapeutic Implications. Antioxidants (Basel) 8 (6), 164. 10.3390/antiox8060164 PMC6617024 31174269

[B60] Qaum T. Xu Q. Joussen A. M. Clemens M. W. Qin W. Miyamoto K. (2001). VEGF-initiated Blood-Retinal Barrier Breakdown in Early Diabetes. Invest. Ophthalmol. Vis. Sci. 42 (10), 2408–2413. 11527957

[B61] Qin Y. Zhai Q. Li Y. Cao M. Xu Y. Zhao K. (2018). Cyanidin-3-O-glucoside Ameliorates Diabetic Nephropathy through Regulation of Glutathione Pool. Biomed. Pharmacother. 103, 1223–1230. 10.1016/j.biopha.2018.04.137 29864902

[B62] Rodríguez M. L. Pérez S. Mena-Mollá S. Desco M. C. Ortega Á. L. (2019). Oxidative Stress and Microvascular Alterations in Diabetic Retinopathy: Future Therapies. Oxid. Med. Cel. Longev. 2019, 4940825. 10.1155/2019/4940825 PMC6878793 31814880

[B63] Rossino M. G. Casini G. (2019). Nutraceuticals for the Treatment of Diabetic Retinopathy. Nutrients 11 (4), 771. 10.3390/nu11040771 PMC6520779 30987058

[B64] Rossino M. G. Dal Monte M. Casini G. (2019). Relationships between Neurodegeneration and Vascular Damage in Diabetic Retinopathy. Front. Neurosci. 13, 1172. 10.3389/fnins.2019.01172 31787868 PMC6856056

[B65] Rübsam A. Parikh S. Fort P. E. (2018). Role of Inflammation in Diabetic Retinopathy. Int. J. Mol. Sci. 19 (4), 942. 10.3390/ijms19040942 PMC5979417 29565290

[B66] Sadri H. Larki N. N. Kolahian S. (2017). Hypoglycemic and Hypolipidemic Effects of Leucine, Zinc, and Chromium, Alone and in Combination, in Rats with Type 2 Diabetes. Biol. Trace Elem. Res. 180 (2), 246–254. 10.1007/s12011-017-1014-2 28409409

[B67] Sasaki M. Ozawa Y. Kurihara T. Kubota S. Yuki K. Noda K. (2010). Neurodegenerative Influence of Oxidative Stress in the Retina of a Murine Model of Diabetes. Diabetologia 53 (5), 971–979. 10.1007/s00125-009-1655-6 20162412 PMC2850533

[B68] Sasaki R. Nishimura N. Hoshino H. Isa Y. Kadowaki M. Ichi T. (2007). Cyanidin 3-glucoside Ameliorates Hyperglycemia and Insulin Sensitivity Due to Downregulation of Retinol Binding Protein 4 Expression in Diabetic Mice. Biochem. Pharmacol. 74 (11), 1619–1627. 10.1016/j.bcp.2007.08.008 17869225

[B69] Semeraro F. Cancarini A. dell'Omo R. Rezzola S. Romano M. R. Costagliola C. (2015). Diabetic Retinopathy: Vascular and Inflammatory Disease. J. Diabetes Res. 2015, 582060. 10.1155/2015/582060 26137497 PMC4475523

[B70] Shi C. Wang P. Airen S. Brown C. Liu Z. Townsend J. H. (2020). Nutritional and Medical Food Therapies for Diabetic Retinopathy. Eye Vis. (Lond) 7, 33. 10.1186/s40662-020-00199-y 32582807 PMC7310218

[B71] Shinoda K. Rejdak R. Schuettauf F. Blatsios G. Völker M. Tanimoto N. (2007). Early Electroretinographic Features of Streptozotocin-Induced Diabetic Retinopathy. Clin. Exp. Ophthalmol. 35 (9), 847–854. 10.1111/j.1442-9071.2007.01607.x 18173414

[B72] Simó R. Stitt A. W. Gardner T. W. (2018). Neurodegeneration in Diabetic Retinopathy: Does it Really Matter? Diabetologia 61 (9), 1902–1912. 10.1007/s00125-018-4692-1 30030554 PMC6096638

[B73] Sinclair S. H. Schwartz S. S. (2019). Diabetic Retinopathy-An Underdiagnosed and Undertreated Inflammatory, Neuro-Vascular Complication of Diabetes. Front. Endocrinol. (Lausanne) 10, 843. 10.3389/fendo.2019.00843 31920963 PMC6923675

[B74] Tang J. Kern T. S. (2011). Inflammation in Diabetic Retinopathy. Prog. Retin. Eye Res. 30 (5), 343–358. 10.1016/j.preteyeres.2011.05.002 21635964 PMC3433044

[B75] Tena N. Martín J. Asuero A. G. (2020). State of the Art of Anthocyanins: Antioxidant Activity, Sources, Bioavailability, and Therapeutic Effect in Human Health. Antioxidants (Basel) 9 (5), 451. 10.3390/antiox9050451 PMC7278599 32456252

[B76] Thounaojam M. C. Powell F. L. Patel S. Gutsaeva D. R. Tawfik A. Smith S. B. (2017). Protective Effects of Agonists of Growth Hormone-Releasing Hormone (GHRH) in Early Experimental Diabetic Retinopathy. Proc. Natl. Acad. Sci. U S A. 114 (50), 13248–13253. 10.1073/pnas.1718592114 29180438 PMC5740669

[B77] Vecino E. Rodriguez F. D. Ruzafa N. Pereiro X. Sharma S. C. (2016). Glia-neuron Interactions in the Mammalian Retina. Prog. Retin. Eye Res. 51, 1–40. 10.1016/j.preteyeres.2015.06.003 26113209

[B78] Vishwanathan R. Chung M. Johnson E. J. (2013). A Systematic Review on Zinc for the Prevention and Treatment of Age-Related Macular Degeneration. Invest. Ophthalmol. Vis. Sci. 54 (6), 3985. 10.1167/iovs.12-11552 23652490

[B79] Wang Y. Huo Y. Zhao L. Lu F. Wang O. Yang X. (2016). Cyanidin-3-glucoside and its Phenolic Acid Metabolites Attenuate Visible Light-Induced Retinal Degeneration *In Vivo* via Activation of Nrf2/HO-1 Pathway and NF-κB Suppression. Mol. Nutr. Food Res. 60 (7), 1564–1577. 10.1002/mnfr.201501048 26991594

[B80] Wen Y. Huo S. Zhang W. Xing H. Qi L. Zhao D. (2016). Pharmacokinetics, Biodistribution, Excretion and Plasma Protein Binding Studies of Acteoside in Rats. Drug Res. (Stuttg) 66 (3), 148–153. 10.1055/s-0035-1555896 26241371

[B81] Wu L. Georgiev M. I. Cao H. Nahar L. El-Seedi H. R. Sarker S. D. (2020). Therapeutic Potential of Phenylethanoid Glycosides: A Systematic Review. Med. Res. Rev. 40 (6), 2605–2649. 10.1002/med.21717 32779240

[B82] Xiao H. Gu Z. Wang G. Zhao T. (2013). The Possible Mechanisms Underlying the Impairment of HIF-1α Pathway Signaling in Hyperglycemia and the Beneficial Effects of Certain Therapies. Int. J. Med. Sci. 10 (10), 1412–1421. 10.7150/ijms.5630 23983604 PMC3752727

[B83] Xiong W. T. Gu L. Wang C. Sun H. X. Liu X. (2013). Anti-hyperglycemic and Hypolipidemic Effects of Cistanche Tubulosa in Type 2 Diabetic Db/db Mice. J. Ethnopharmacol. 150 (3), 935–945. 10.1016/j.jep.2013.09.027 24095831

[B84] Xu H. Z. Le Y. Z. (2011). Significance of Outer Blood-Retina Barrier Breakdown in Diabetes and Ischemia. Invest. Ophthalmol. Vis. Sci. 52 (5), 2160–2164. 10.1167/iovs.10-6518 21178141 PMC3080181

