# Supplementary figures and images for: Preventive Efficacy of an Antioxidant Compound on Blood Retinal Barrier Breakdown and Visual Dysfunction in Streptozotocin-Induced Diabetic Rats
Source: Front Pharmacol. 2022 Jan 3;12:811818. doi: 10.3389/fphar.2021.811818 (PMC8762314; doi:10.3389/fphar.2021.811818)

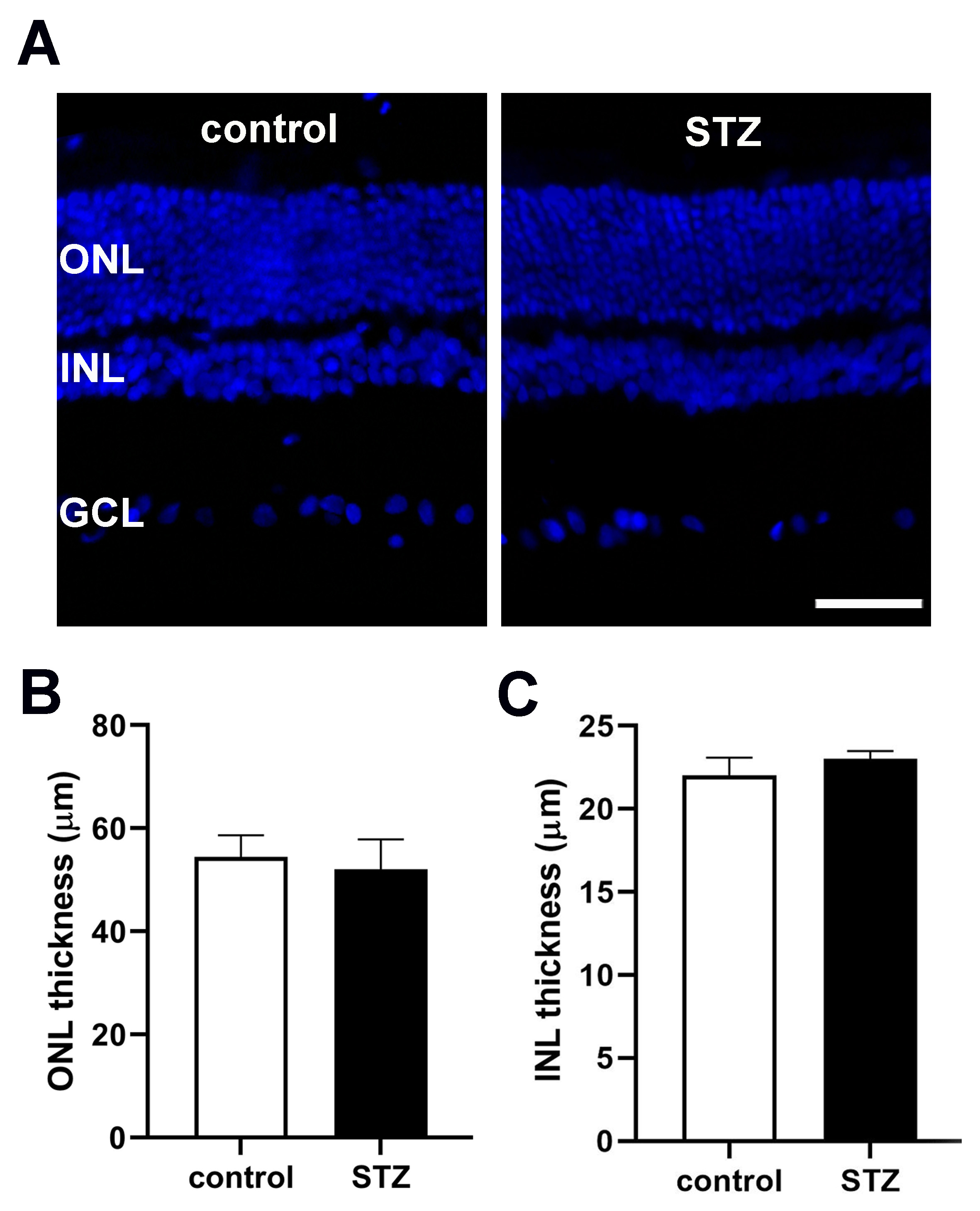

Supplement: Supplementary file 1 [file Image3.JPEG]

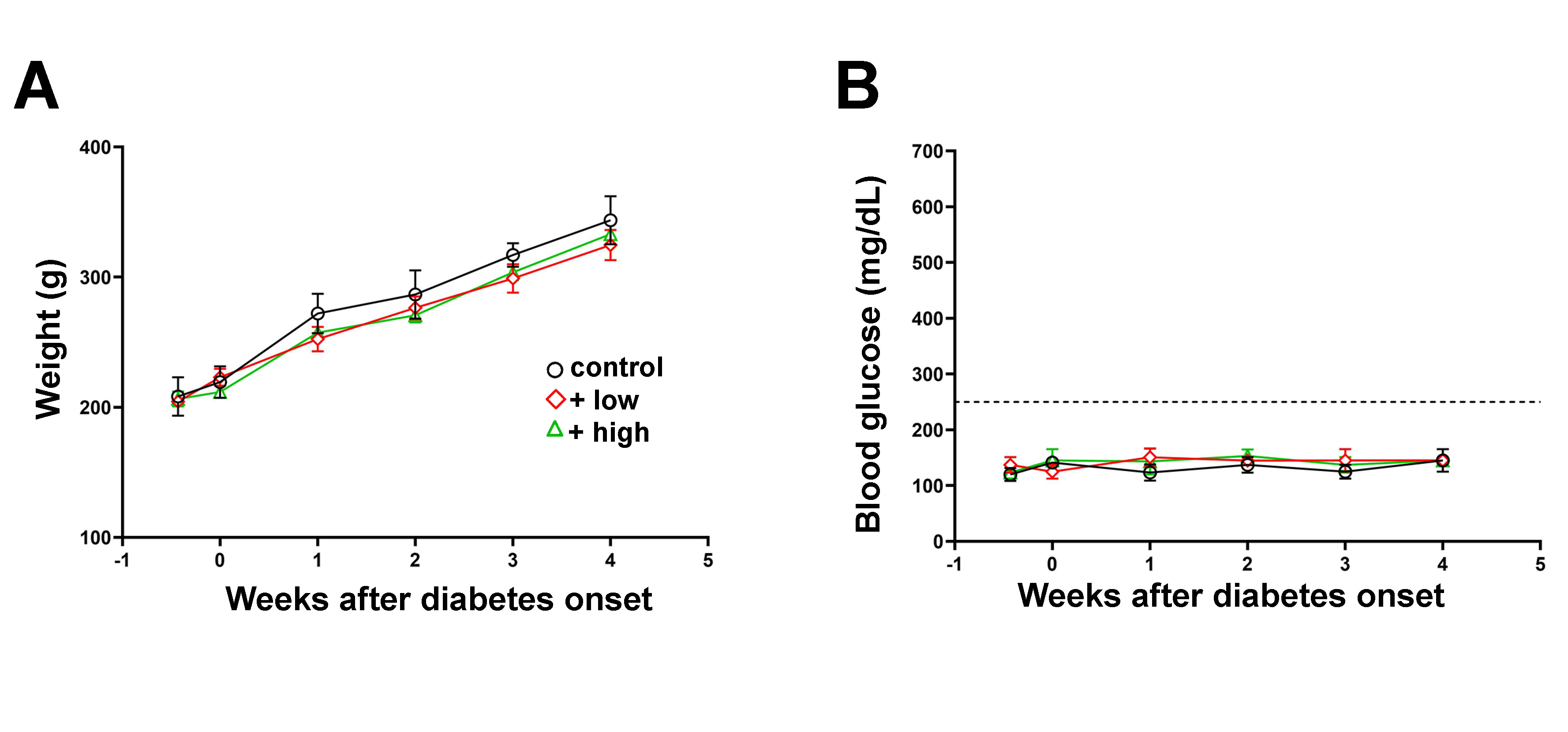

Supplement: Supplementary file 2 [file Image1.JPEG]

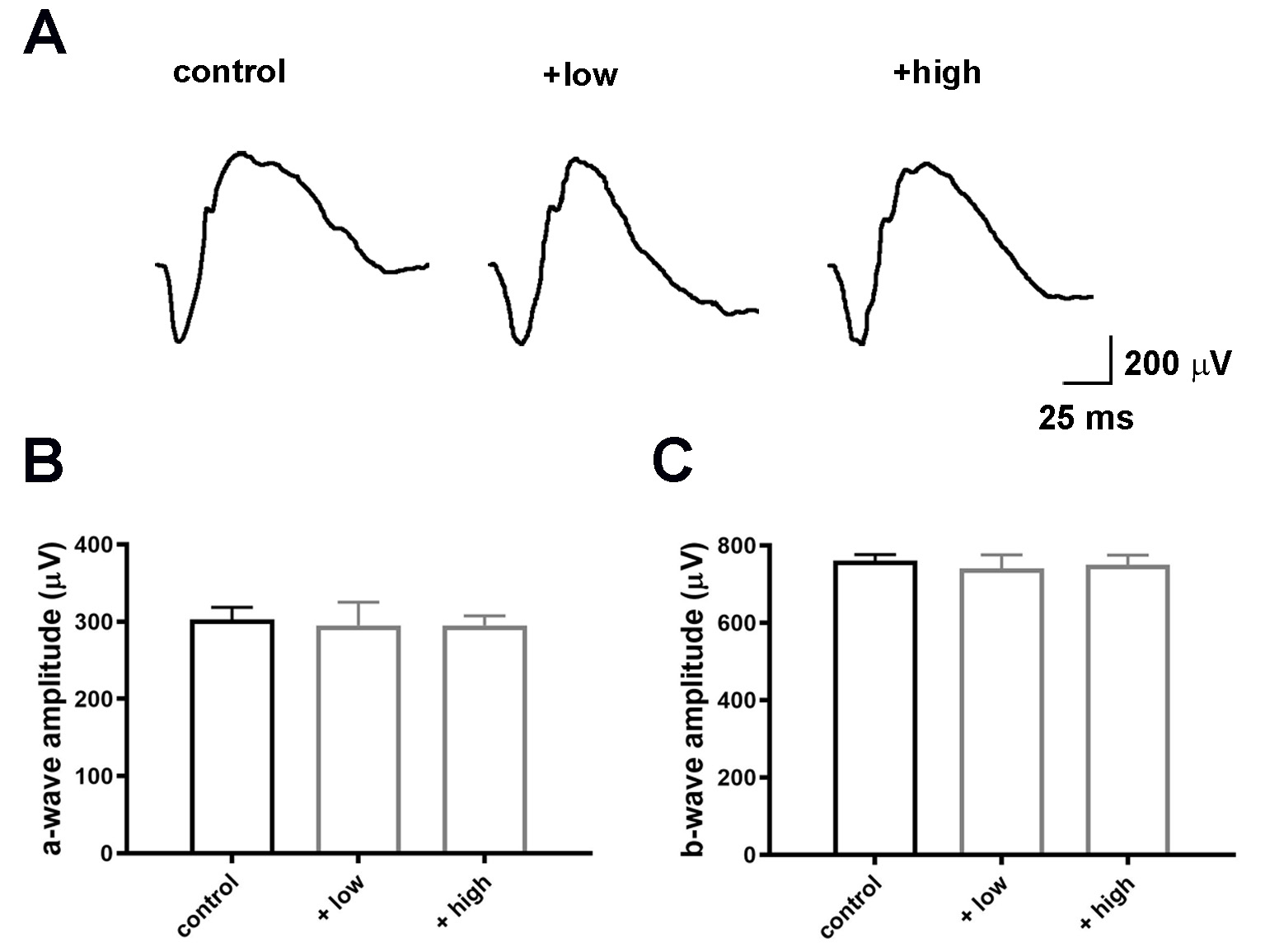

Supplement: Supplementary file 3 [file Image2.JPEG]
